# Glycogen synthase kinase 3-β inhibition induces lymphangiogenesis through β-catenin-dependent and mTOR-independent pathways

**DOI:** 10.1371/journal.pone.0213831

**Published:** 2019-04-09

**Authors:** Benjamin Stump, Shikshya Shrestha, Anthony M. Lamattina, Pierce H. Louis, Woohyun Cho, Mark A. Perrella, Xingbin Ai, Ivan O. Rosas, Florence F. Wagner, Carmen Priolo, Jonathan Astin, Souheil El-Chemaly

**Affiliations:** 1 Division of Pulmonary and Critical Care Medicine, Brigham and Women’s Hospital, Harvard Medical School, Boston, Massachusetts, United States of America; 2 Stanley Center for Psychiatric Research, Broad Institute of Massachusetts Institute of Technology and Harvard University, Cambridge, Massachusetts, United States of America; 3 Department of Molecular Medicine and Pathology, School of Medical Sciences, The University of Auckland, Auckland, New Zealand; Medical College of Georgia at Augusta University, UNITED STATES

## Abstract

Lymphatic vessels play an important role in health and in disease. In this study, we evaluated the effects of GSK3-β inhibition on lung lymphatic endothelial cells *in vitro*. Pharmacological inhibition and silencing of GSK3-β resulted in increased lymphangiogenesis of lung lymphatic endothelial cells. To investigate mechanisms of GSK3-β-mediated lymphangiogenesis, we interrogated the mammalian/mechanistic target of rapamycin pathway and found that inhibition of GSK3-β resulted in PTEN activation and subsequent decreased activation of AKT, leading to decreased p-P70S6kinase levels, indicating inhibition of the mTOR pathway. In addition, consistent with a negative role of GSK3-β in β-catenin stability through protein phosphorylation, we found that GSK3-β inhibition resulted in an increase in β-catenin levels. Simultaneous silencing of β-catenin and inhibition of GSK3-β demonstrated that β-catenin is required for GSK3-β-induced lymphangiogenesis.

## Introduction

Lymphatic vessels play critical roles in health and disease by maintaining tissue homeostasis through their involvement in draining macromolecules and immune cell trafficking [1]. The role and importance of lymphatics in cancer biology, and the importance of blocking lymphangiogenesis in cancer metastasis have long been recognized (for review [2]) High-throughput drug screens have identified compounds that would inhibit lymphangiogenesis [3–5]. However, the therapeutic benefit of stimulation of lymphatic vessel formation or function has more recently been recognized in heart [6], lung [7–9], and other solid organs (for review [10]). Lymphangiogenesis is induced by a variety of growth factors, including vascular endothelial growth factor (VEGF)-C and VEGF-D which signal through their canonical receptor VEGF receptor (VEGFR)-3 (for review [11, 12]). However, few compounds have been shown to induce lymphangiogenesis *in vitro* or *in vivo* [13, 14].

Glycogen synthase kinase (GSK)-3 is a kinase with wide biological roles [15]. Activated GSK3-β phosphorylates β-catenin, thereby targeting it for degradation [16]. Furthermore, GSK3-mediated phosphorylation activates tuberin which leads to inactivation of the mammalian/mechanistic target of rapamycin (mTOR) pathway [17].

β-catenin has been shown to play important roles in lymphatic vessel patterning [18], and that WNT ligands from myeloid cells are important for lymphatic vessel development [19]. More recent evidence demonstrates that β-catenin forms a complex with prospero homeobox protein 1 (PROX1), a master regulator of lymphatic lineage, to augment β-catenin signaling [20]. Intriguingly, however, LECs express less β-catenin than blood endothelial cells [21]. On the other hand, mTOR is a common pathway for many lymphangiogenic growth factors, and mTOR blockade results in inhibition of lymphangiogenesis [22–24]. In addition, rapamycin, an mTOR inhibitor, promotes VEGFR-3 degradation, decreasing LECs’ ability to respond to lymphangiogenic growth factors [25]. Therefore, GSK3 inhibition could lead to lymphangiogenesis either by stabilizing β-catenin or through activation of the mTOR pathway.

Our laboratory and others have shown the importance of induction of lymphangiogenesis in lung disease [7, 9, 26, 27]. Using human lung lymphatic endothelial cells, the aim of our studies was to investigate the potential role of GSK3-β and to identify downstream pathways involved in the regulation of lymphangiogenesis *in vitro*. Our findings provide evidence that GSK3-β inhibition induces lymphangiogenesis through mTOR-independent and β-catenin-dependent pathways.

## Materials and methods

### Cells and reagents

Human lung microvascular endothelial cells from a single donor (HMVEC-L Cat # CC-2527, Lonza, Rockville, MD) were used for *in vitro* assays. Cells were maintained in microvascular cell culture media with growth supplements (Cell Applications Inc., San Diego, CA), unless stated otherwise. Cells were incubated at 37° C in a humidified 5% CO_2_ chamber. SB216763 (Sigma, St. Louis, MO) was reconstituted in DMSO and stored at -20°C per manufacturer directions. SB216763 dosing for all experiments was 1μM unless stated otherwise. BRD3731, a novel selective inhibitor of GSK-3β, was generously provided by Dr. Florence Wagner (Broad Institute, Cambridge, MA) and was also reconstituted in DMSO. Control groups were treated with an equivalent amount of vehicle (DMSO).

### Cell proliferation

Cells between passage 3 and 4 were seeded into wells of a 96 well plate at a density of 1000 cells per well and allowed to incubate in 100 μL of full culture media for 24 hours. The culture media was then replaced with microvascular endothelial cell basal media without growth supplements overnight. Following starvation, cells were treated with basal media supplemented with 1% FBS and either DMSO (vehicle) or SB216763. The optimal dosing of SB216763 after dose titration experiments was 1μM. Cell proliferation was determined using CyQuant^™^NF assay kit (Invitrogen, Carlsbad, CA) per the manufacturer instructions. Fluorescence intensity was determined using a Biotek Synergy HT microplate reader (Biotek, Winooski, VT).

### Cell migration

Cells were seeded into the top chamber of a Cultrex^®^ Cell Invasion/Migration Chamber (Trevigen, Gaithersburg, MD) at a density of 5 X 10^4^ cells per well in 50 μL of basal media with either SB216763 1μM or an equivalent amount of DMSO. Lower chambers were filled with 150 μL of basal media. The migration chamber was incubated for 24 hours. The chambers were aspirated and washed per manufacturer instructions using supplied wash buffer. A cell dissociation solution with Calcein-AM (Corning, Corning, NY) was then placed in the lower chambers and the migration chamber was placed back in the incubator for 30 minutes. The top chamber was then removed, and fluorescence intensity was determined using a Biotek Synergy HT microplate reader.

### Matrigel tubulation

Growth factor reduced Matrigel (Corning, Corning, NY) was used for all tubulation experiments [28]. As per manufacturer protocol, 289 μL of Matrigel was placed in wells of a 24 well plate followed by incubation at 37° C for 1 hour to prepare the matrix. Cells were seeded at a density of 1.2 X10^5^ cells per well in 300 μL of basal media with SB216763 1 μM or an equivalent amount of DMSO. Cells were incubated for 16–18 hours followed by aspiration of the media without disturbing the Matrigel. Wells were gently washed twice with HBSS 1X twice followed by incubation with Calcein-AM 8μg/ml) in HBSS 1X for 30 minutes. Wells were then washed twice with HBSS 1X. 5–10 non-overlapping 4X fluorescent images were obtained using a Nikon Eclipse TS110 microscope and Nikon DS-Ri2 camera. Images were analyzed using ImageJ (NIH, Bethesda, MD) and a previously designed Angiogenesis Analyzer plug-in [29]. Data used for analysis, including number of meshes and total mesh, are per image.

### Immunoblotting

Cells were incubated in 100 mm dishes in full cell culture media until 70% confluent. Full culture media was then replaced with basal media without growth supplements and incubated overnight. Cells were then treated with basal media supplemented with 1% FBS and either SB216763 1μM or an equivalent amount of DMSO. After 48 hours, cells were washed with sterile PBS and protein lysates were harvested with RIPA Buffer. Proteins were separated on NuPAGE Bis-Tris 4–12% gels (Thermo Fisher Scientific, Waltham, MA) and transferred to a nitrocellulose membrane, which was then exposed to the indicated primary antibodies (Table 1). Each protein of interest was then detected with HRP-conjugated goat anti-rabbit or anti-mouse IgG (H+L) antibody (1:1000, Thermo Fisher Scientific), and visualized using SuperSignal West Pico PLUS Chemiluminescent Substrate or SuperSignal^™^ West Femto Maximum Sensitivity Substrate (Thermo Fisher Scientific). The same process was repeated with cells treated with BRD3731 5μM or an equivalent amount of DMSO for western blot analysis.

**Table 1:** Antibodies used in these studies.

Antigen	Source
phospho P70S6K (Thr 389)	Cell Signalling Technology 9234
Total P70S6K	Cell Signalling Technology 2708
phospho-β-catenin (Ser33/37/Thr41)	Cell Signalling Technology 9561
β-catenin	Cell Signalling Technology 8480
phospho-PTEN (T366)	ABCAM AB109454
PTEN	Cell Signalling Technology 9559
β-Actin	ABCAM AB8226
phospho-AKT (Ser473)	Cell Signalling Technology 9271
AKT	Cell Signalling Technology 4685
GSK3-β	Cell Signalling Technology 9315
GSK3-α	Cell Signalling Technology 4337

### RNA interference

Predesigned siRNA targeting GSK-3β, β-Catenin, and Mission siRNA Universal Negative Control were obtained from Sigma (sequences for GSK-3β and β-Catenin siRNA can be found in Table 2). Transfection was performed using a 6nM concentration of siRNA in Lipofectamine RNAiMAX (Thermo Fisher Scientific). Transfection media containing SiRNA, Lipofectamine, and Opti-MEM (Thermo Fisher Scientific) was removed 6 hours following initial transfection and replaced with full cell culture media. Cells were incubated for a total of 48 hours after initial transfection and were subsequently harvested for protein collection or counted and placed on Matrigel for evaluation of tubulation as described above. Similarly, protein lysates were prepared, and immunoblotting performed as described above. Primary antibodies against phospho- β-catenin (Ser33/37/Thr41), β-catenin, GSK3-β, phospho P70S6K (Thr 389), Total P70S6K, phospho-PTEN (T366), PTEN, and β-Actin (Table 1**)** were utilized.

**Table 2:** siRNA sequences human.

SiRNA	SA Sequence reference #	Sequence (5'-3')
*GSK3-β* NM_002093	SASI_Hs01_00192106	Sense GGACUAUGUUCCGGAAACA[dT][dT]
	SASI_Hs01_00192106_AS	Anti-sense UGUUUCCGGAACAUAGUCC[dT][dT]
*β-catenin* NM_001904	SASI_Hs01_00117959	Sense GAAUGAAGGUGUGGCGACA[dT][dT]
	SASI_Hs01_00117959_AS	Anti-sense UGUCGCCACACCUUCAUUC[dT][dT]

All original uncut blots are included in S1 Fig.

### Statistical analysis

Cell proliferation, migration, tubulation, and immunoblotting experiments to evaluate the effects of SB216763 were repeated at least three times as were the immunoblotting experiments using BRD3731. Genetic knockdown experiments evaluating the effects of GSK-3β and β-Catenin siRNA on Matrigel tubulation were repeated twice. Data are expressed as means (± SEM). Statistical significance between groups was determined by a p value less than 0.05 using *t* test or ANOVA. Data analysis was conducted using Graphpad Prism 7 (GraphPad Software Inc., San Diego, CA).

## Results

### Treatment with SB216763 induces lymphatic endothelial cell proliferation, migration and network formation *in vitro*

**Fig 1:**
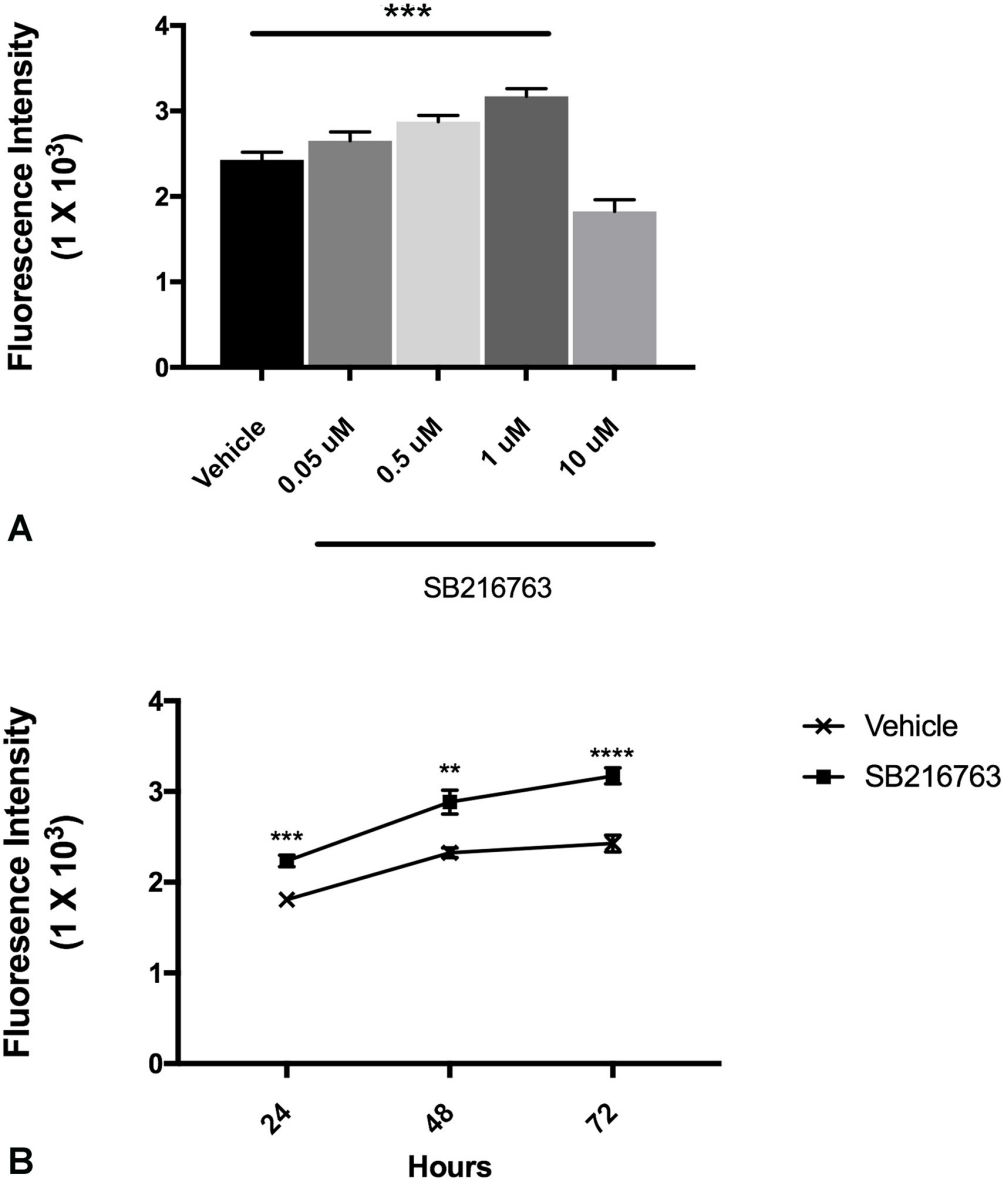
SB216763 induces lymphatic endothelial cell proliferation in a time and dose-dependent manner. A) Primary human lung LECs in 1% FBS were incubated with vehicle or SB216763 at increasing concentration for 48 hours. B) LECs incubated with SB216763 (1μM) or vehicle (DMSO) demonstrated a time-dependent growth. Depicted are Mean ± SEM of 8 replicates. * *P* < 0.05, **P < 0.01, ***P < 0.001 by ANOVA. Results were repeated at least twice.

**Fig 2:**
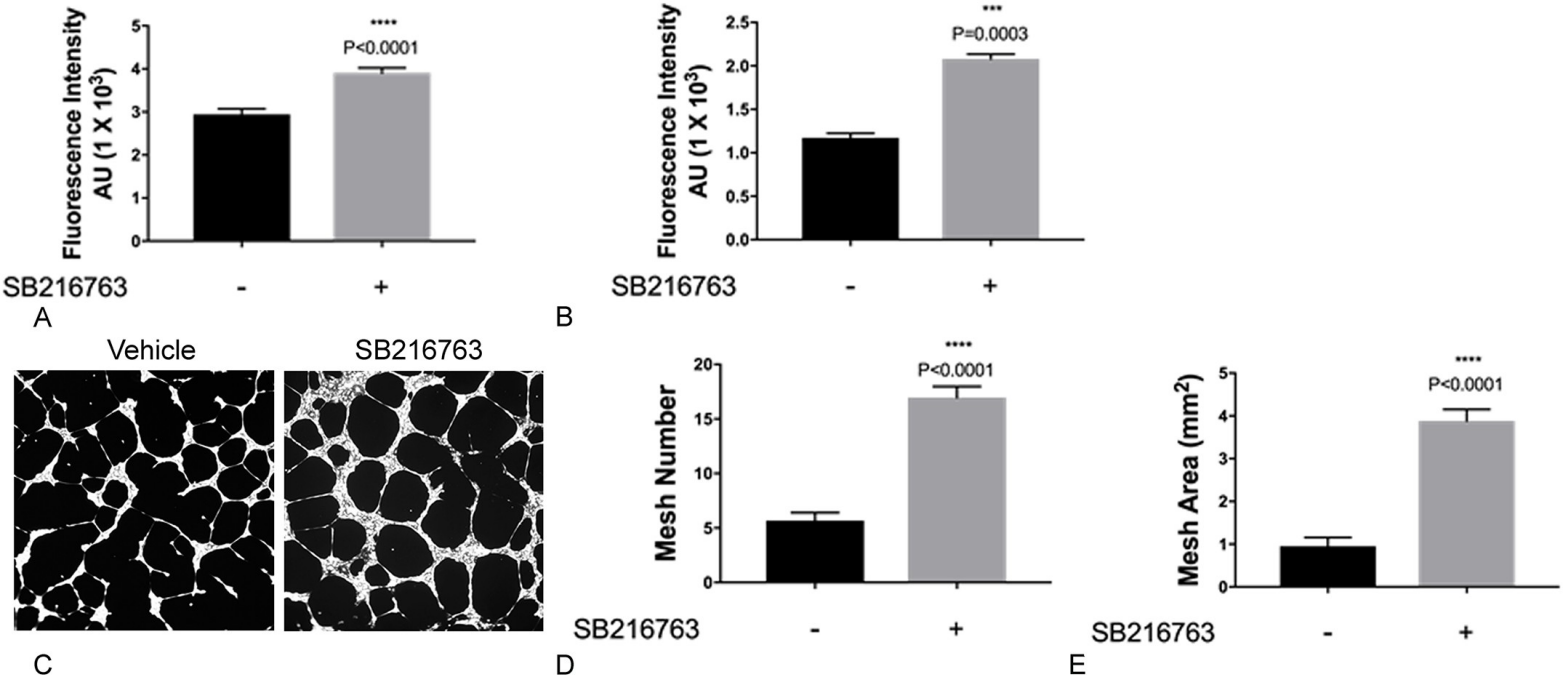
GSK3 inhibition induces proliferation, migration and network formation of primary human lung lymphatic endothelial cells. A) Primary human lung LECs in 1% FBS were incubated with SB216763 (1μM) or vehicle (DMSO) alone. Cell proliferation was determined at 48h after treatment using CyQUANT assay (**A**). Effect of SB216763 (1μM) on the migration of primary LECs was evaluated in transwell migration assays (B). Effect of SB216763 (1μM) on tube formation of primary LECs. An equal number of LECs were seeded onto growth factor-reduced Matrigel at a density of 1.2x10^4^ cells/well and incubated with either SB216763 (1μM) or vehicle alone (DMSO). After 16 hours, cells were labeled with Calcein-AM and random 4X fluorescent images were taken (C) and the number of meshes (D) and mesh area (E) per image were determined using ImageJ (NIH) Angiogenesis Analyzer plugin. Results are expressed as fold change compared to vehicle control. Data represent Mean ± SEM of a single experiment. All experiments were repeated a minimum of three times. *P < 0.05, ** *P* < 0.01, *** *P* < 0.001 by t-test.

To examine the effects of GSK3 inhibition on LEC proliferation, migration and tube formation *in vitro*, we first performed concentration- and time- response curves and determined that a concentration of 1μM at 48h is optimal dose and time for evaluation of the effects of SB216763 on lung lymphatic endothelial cells (Fig 1A and 1B). SB216763 induced LEC proliferation as measured using a cell permeant DNA-binding fluorescent dye (Fig 2A). We next, tested the effects of SB216763 on LEC migration. There was a modest but significant increase in LEC migration in a Boyden chamber (Fig 2B). Next, we examined the effects of SB216763 on LEC network formation in Matrigel. SB216763 (1μM) promoted a ~ 4-fold increase in tube formation as measured by the number of meshes or mesh area compared to vehicle control (Fig 2C, 2D and 2E). Taken together, these data support our hypothesis that an inhibitor of GSK3 enhanced all aspects of LEC behavior characteristic of lymphangiogenesis *in vitro*.

### Treatment with SB216763 results in inactivation of the mTOR pathway *in vitro*

**Fig 3:**
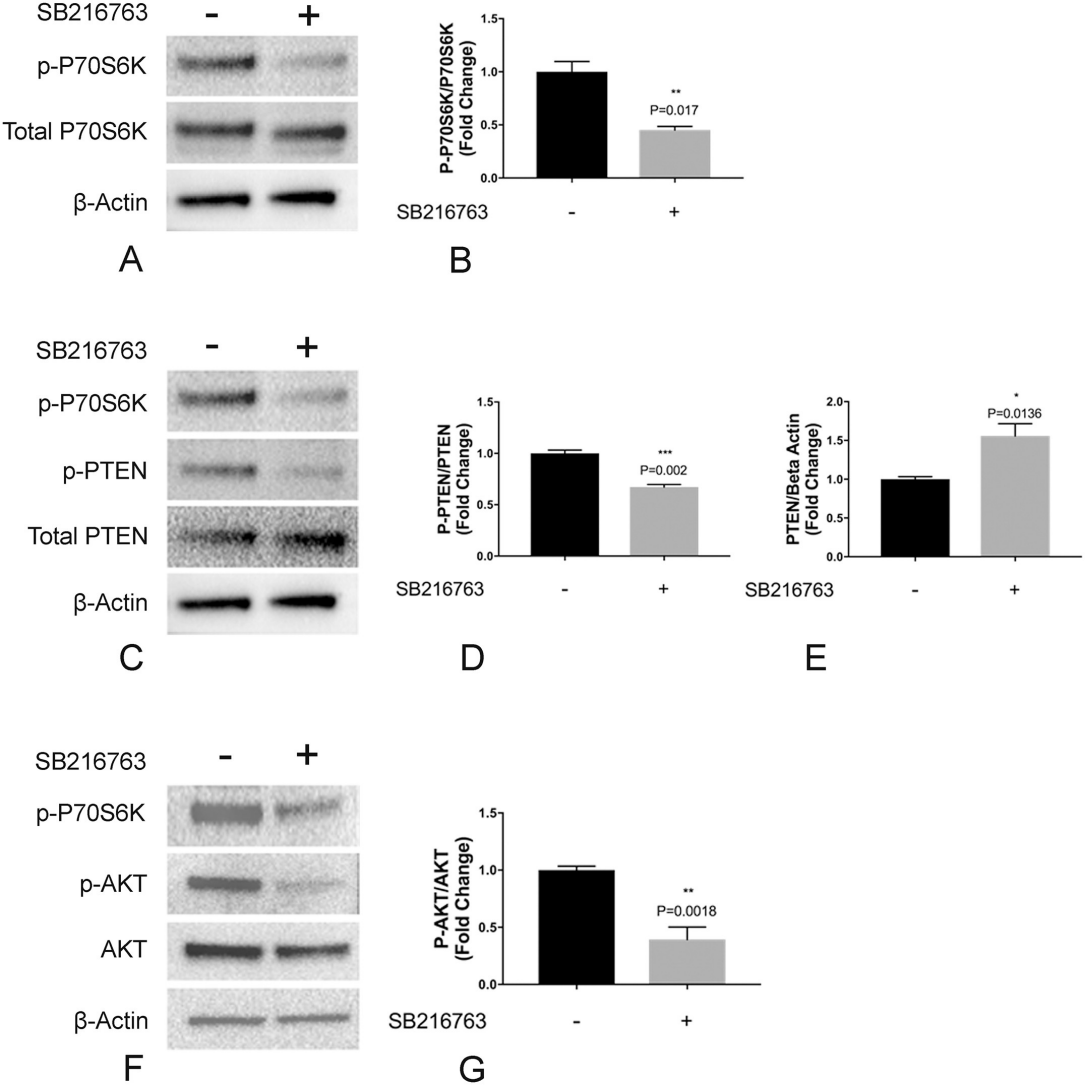
GSK3 inhibition resulted in inactivation of the mTOR pathway in primary lung lymphatic endothelial cells. Primary lung lymphatic endothelial cells were treated with either SB216763 (1μM) or vehicle control (DMSO) for 48h. Equal amounts of protein from whole cell lysates were analyzed by Western blotting with antibodies against phosphorylated and total P70S6kinase (A), phosphorylated and total PTEN (C), phosphorylated and total Akt (F) and β-actin. Ratio of phophospho-P70S6kinase and total P70S6Kinase (B), phosphorylated and total PTEN (D), and total PTEN/ β-actin (E) and phospho-AKT and total AKT (G), expressed as the fold-change relative to unstimulated control. Data represent means ± SEM of three independent experiments. * *P* < 0.05, **P < 0.01, ***P < 0.001 by t-test.

GSK3-β has been shown to phosphorylate tuberin, leading to inactivation of the mTOR pathway [17]. To test the hypothesis that GSK3 inhibition leads to enhanced lymphangiogenesis *in vitro* through activation of the mTORC1 pathway, LECs were incubated with SB216763 (1μM) and resulting effects on the mTORC1 pathway were evaluated by immunoblotting for p-P70S6Kinase. Surprisingly, and contrary to previously published data in HEK293 cells [17], we found that inhibition of GSK3 in LECs resulted in decreased p-P70S6K, suggesting inhibition of the mTOR pathway (Fig 3A and 3B).

The mTOR pathway and its complex regulatory molecules have been extensively studied [30]. Phosphatase and tensin homolog (PTEN) regulates AKT which in turn regulates phospho-S6 [30]. To investigate mechanisms of GSK3-induced inhibition of mTOR, we evaluated the effects of SB216763 on AKT and PTEN activation. We found that treatment resulted in decreased phosphorylation of PTEN at residue T366, a phosphorylation site which tags PTEN for degradation [31]. The SB216763-induced dephosphorylation of T366 results in increased PTEN levels (Fig 2C, 2D and 2E). These effects of GSK3 inhibition on PTEN resulted in decreased phosphorylation and inactivation of AKT (Fig 3F and 3G), with its known inhibitory effects on activation of the mTOR pathway [30, 32]. Taken together our data show that the observed effects on the mTOR pathway are driven by the effects of SB216763 on PTEN and its downstream targets (Fig 3F and 3G).

### Treatment with SB216763 prevents β-catenin phosphorylation and induces its stabilization

**Fig 4:**
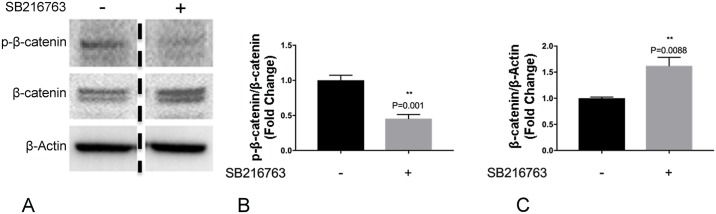
GSK3 inhibition decreases β-catenin phosphorylation and increases β-catenin levels in primary lung lymphatic endothelial cells. Primary human lung lymphatic endothelial cells were treated with either SB216763 (1μM) or vehicle control (DMSO) for 48h. Equal amounts of protein from whole cells lysates were analyzed by western blotting with antibodies against phosphorylated and total β-catenin and β-actin (A). Ratio of phosphorylated and total β-catenin (B), and total β-catenin/β-actin (C) expressed as fold change relative to control. Data represent means ± SEM of three independent experiments. **P <0.01 by t-test.

β-catenin is a direct phosphorylation target of GSK [33], and inhibition of GSK3-β results in decreased phosphorylation and stabilization of β-catenin [34]. To examine the effects of SB216763 on β-catenin in LECs, LECs were treated with either SB216763 (1μM) or vehicle control. Treatment with SB216763 resulted in decreased phosphorylation of β-catenin and increased protein levels (Fig 4A, 4B and 4C), suggesting that the effects of GSK3-β on lymphangiogenesis *in vitro* are driven by increased β-catenin stability.

### Effects of GSK-3β inhibition on mTORC1 and β-catenin pathways are dependent on its kinase activity

**Fig 5:**
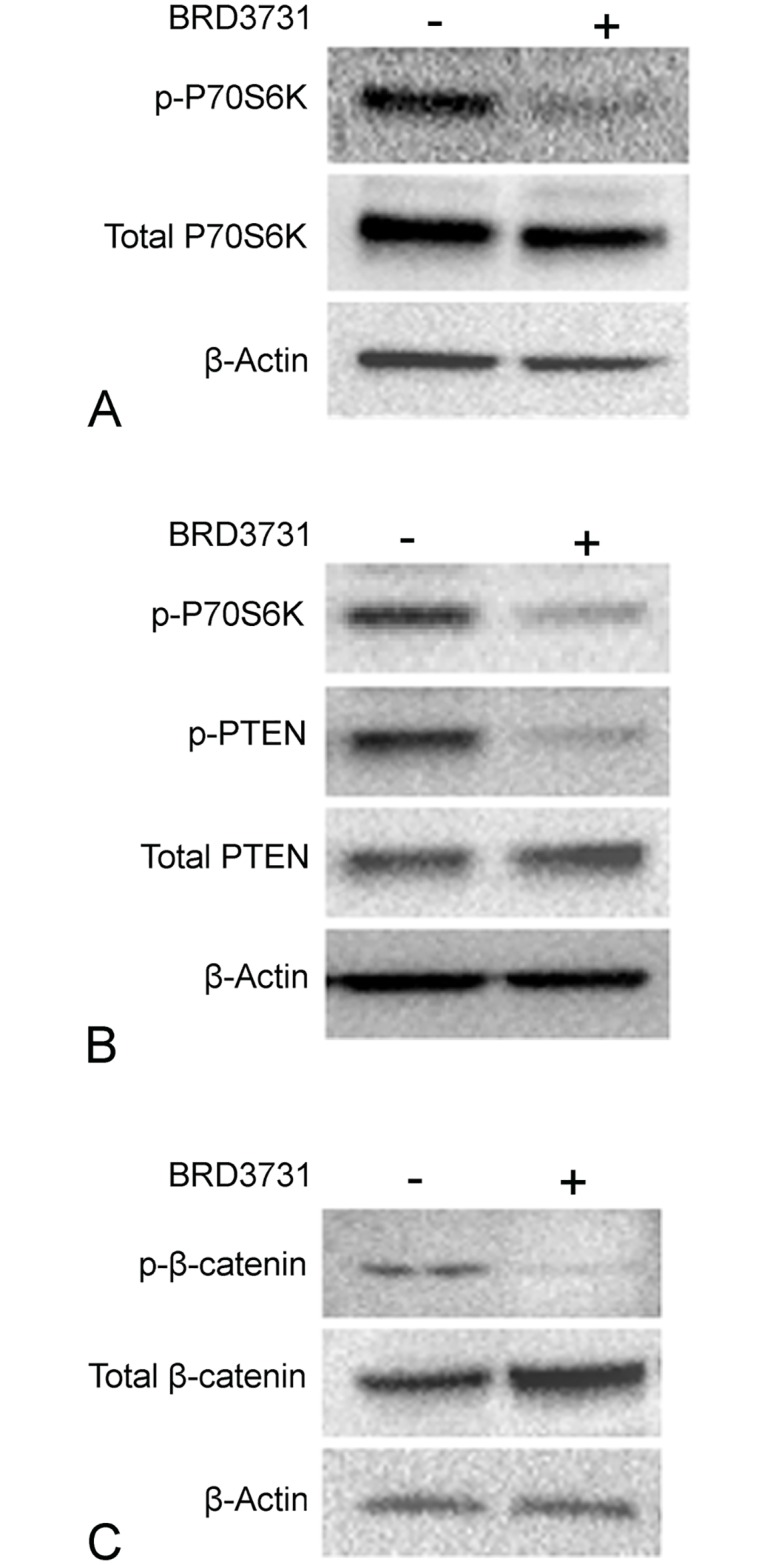
Effects of GSK3-β inhibition are due to inhibition of its kinase activity. Primary human lung lymphatic endothelial cells were treated with either BRD3731 (5μM) or vehicle control (DMSO) for 48h. Equal amounts of protein from whole cells lysates were analyzed by western blotting with antibodies against phosphorylated and total P70S6kinase (A), phosphorylated and total PTEN (B), phosphorylated and total β-catenin (C) and β-actin. Depicted are representative blots from 3 independent experiments.

SB216763 is a potent inhibitor of GSK3-β, but equipotently inhibits its paralog GSK3-α [35]. SB216763 inhibits the kinase activity of GSK3, and recent reports suggest a different potential mechanism of action related to re-arrangement of cellular distribution of GSK3-β [36]. To examine the hypothesis that the effects of SB216763 on the mTORC1 and β-catenin pathways are due to the selective inhibition of GSK3-β and its kinase activity, we used BRD3731, a newly developed paralog selective GSK3-β kinase inhibitor, which has ~6-fold selectivity to GSK3-β compared to GSK3-α [37]. Treatment with BRD3731 resulted in decreased activation of the mTOR pathway (Fig 5A), decreased phosphorylation and increased PTEN levels (Fig 5B), decreased phosphorylation of β-catenin and increased β-catenin stability (Fig 5C). Taken together, these data demonstrate that inhibition of GSK3-β kinase activity in LECs results in enhanced β-catenin levels and decreased activation of the mTOR pathway.

### Effects of silencing GSK3-β on LEC network formation *in vitro*

**Fig 6:**
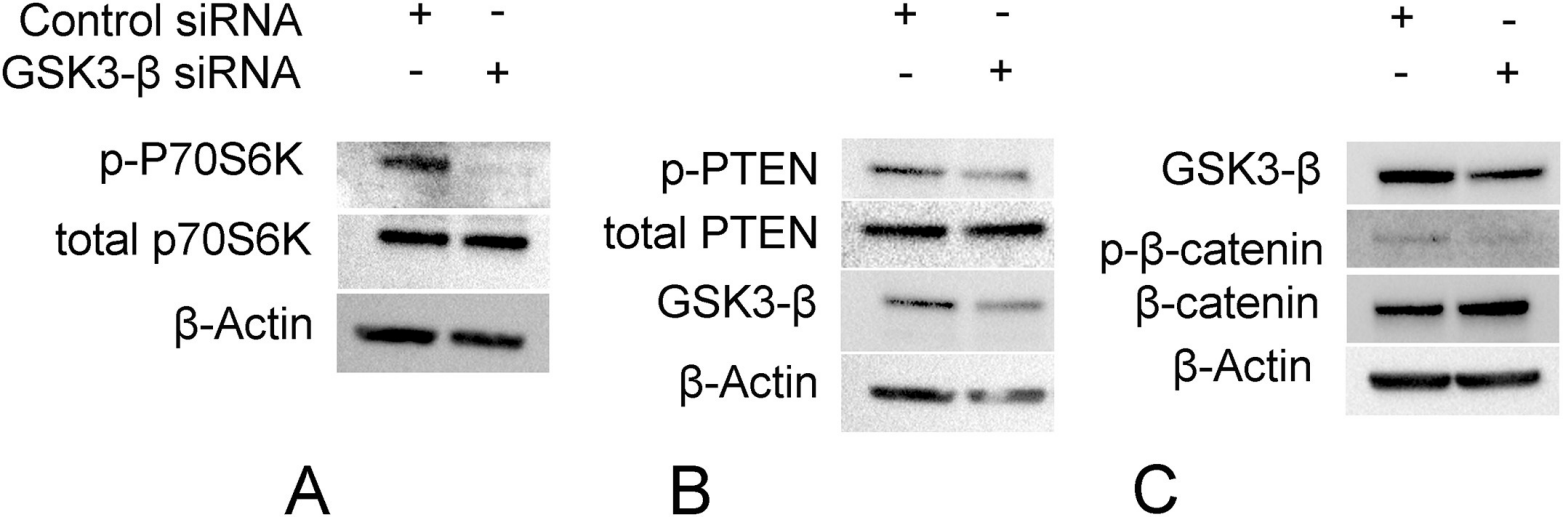
Silencing GSK3-β results in inactivation of the mTOR pathway and activation of the β-catenin pathway in primary lymphatic endothelial cells. Lung lymphatic endothelial cells were transfected with control scrambled siRNA (control siRNA) or GSK3-β siRNA for 48 hours. Equal amounts of protein from whole cell lysates were analyzed by western blotting with antibodies against phosphorylated and total P70S6kinase and β-actin. Same lysates were analyzed on a different membrane for phosphorylated and total PTEN, GSK3-β, β-actin (A) and against phosphorylated and total β-catenin (B).

Finally, and to confirm the effects of GSK3-β inhibition on lung LECs, we used small interference RNA to silence GSK3-β. Silencing GSK3-β resulted in decreased activation of the mTOR pathway as demonstrated by decreased p-P70S6K and p-PTEN (Fig 6A), as well as decreased phosphorylation of β-catenin with an increase in its stability (Fig 6B).

**Fig 7:**
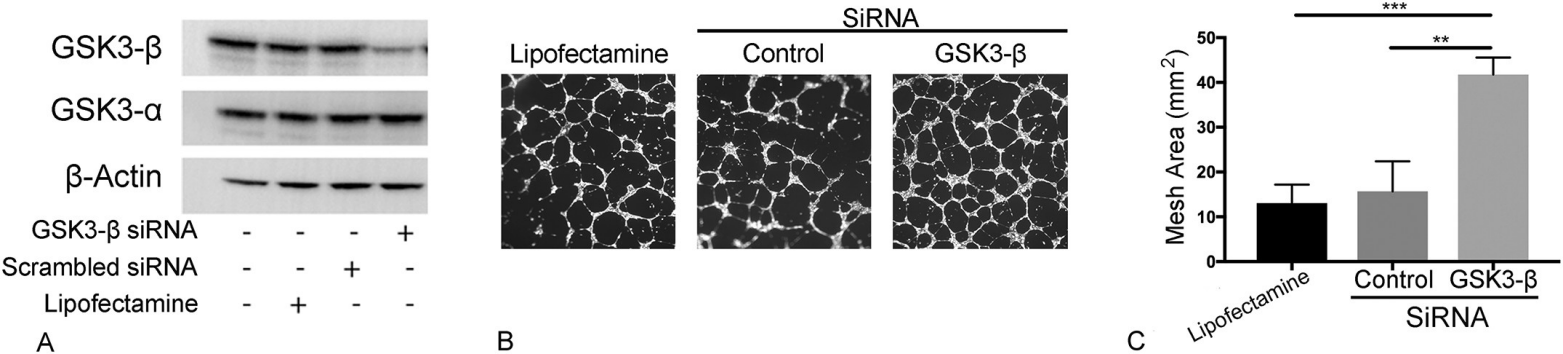
Silencing GSK3-β promotes lymphatic network formation *in vitro*. Lung lymphatic endothelial cells were treated with vehicle (lipofectamine), or transfected with control scrambled siRNA (control siRNA) or GSK3-β siRNA for 48 hours. Equal amounts of protein from whole cell lysates were analyzed by western blotting with antibodies against GSK3-β, GSK3-α and β-actin (A). LECs were treated with either lipofectamine, control siRNA or GSK3-β siRNA for 48h were placed on Matrigel at a density of 1.2x10^4^ cells/well for 16h. Cells were labeled with Calcein-AM. Random 4X images were then obtained (B) and mesh area analyzed with ImageJ Angiogenesis Analyzer plugin (C). Results are expressed as mean ± SEM for one experiment. *P <0.05 by one-way ANOVA. Experiment was repeated once.

To directly study the effects of GSK3-β on LEC network formation *in vitro*, we silenced GSK3-β using small interference RNA which resulted in ~ 65% reduction in GSK3-β but no change in GSK3-α levels (Fig 7A). Silencing GSK3-β resulted in increased mesh formation in Matrigel assay (Fig 7B), demonstrating that with genetic manipulation that GSK3-β is an important regulatory pathway in lung LECs and recapitulating the pharmacological modulation results above.

### Silencing β-catenin abrogates the effects of GSK3-β inhibition on LEC network formation *in vitro*

**Fig 8:**
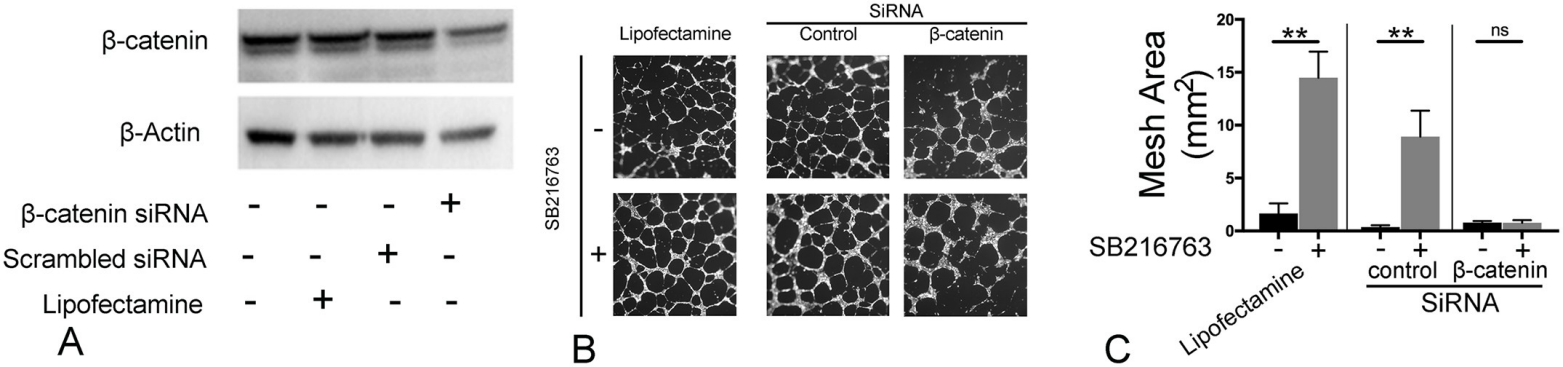
Silencing β-catenin abrogates the effects of GSK3-β inhibition on lymphatic endothelial cell network formation *in vitro*. Lung lymphatic endothelial cells were treated with vehicle (lipofectamine), or transfected with control scrambled siRNA (control siRNA) or β-catenin siRNA for 48h. Equal amounts of protein from whole cell lysates were analyzed by western blotting with antibodies against β-catenin and β-actin (A). LECs were treated with either lipofectamine, control siRNA or β-catenin siRNA for 48h were placed on Matrigel at a density of 1.2X10^4^ cells/well for 16h and then treated with vehicle (DMSO) or SB216763 (1μM). Cells were then labeled with Calcein-AM (8μg/ml). Random 4X images were then obtained (B) and mesh area analyzed with ImageJ Angiogenesis Analyzer plugin (C). Results are expressed as mean ± SEM for one experiment. *P <0.05 by t-test between same group. Experiment was repeated once.

To determine if GSK3-β inhibition drives lymphangiogenesis through β-catenin-dependent pathways, we silenced β-catenin with small interference RNA, causing subsequent decrease in β-catenin levels (Fig 8A). Cells treated with β-catenin siRNA failed to respond to GSK3-β inhibition (SB216763 1μM) as compared to the vehicle or control siRNA-treated lung LECs (Fig 8B and 8C). Taken together, these data show that *in vitro*, GSK3-β-inhibition drives lymphangiogenesis through β-catenin-dependent pathways.

## Discussion

**Fig 9:**
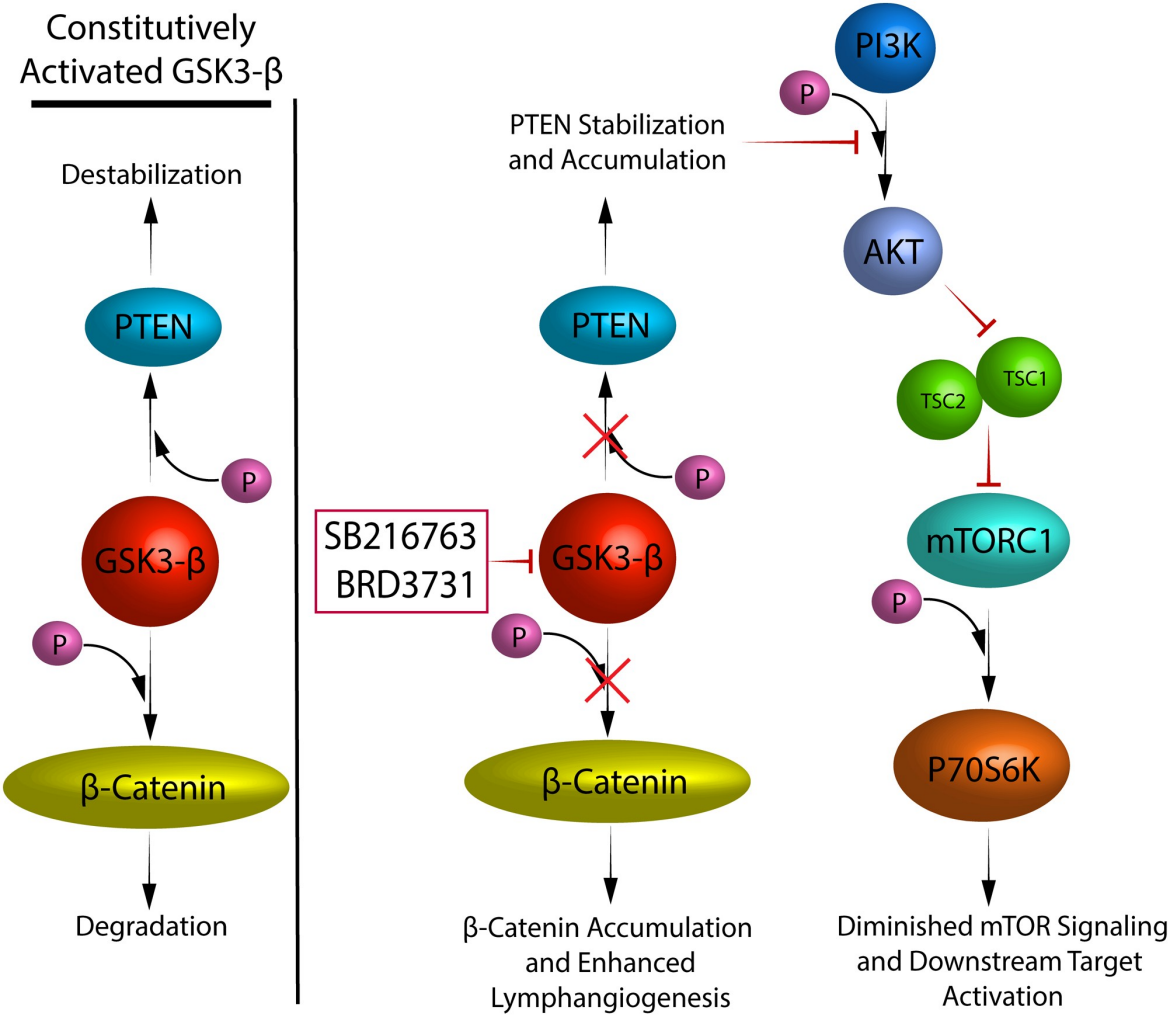
Schematic representation of how GSK3-β regulates lymphangiogenesis.

Lymphatic vessels are essential for maintenance of tissue homeostasis, and if compromised can result in profound changes in tissue edema, immune cell trafficking and fibrosis [6–8, 26, 27]. Few known stimulators of lymphangiogenesis have been described to date. Our results provide new insights on the role of GSK3-β as a regulator of lymphangiogenesis. We have shown that GSK3-β inhibition resulted in increased LEC migration and proliferation and enhanced tubulation *in vitro*, all characteristics of lymphangiogenesis. In addition, we have shown that these effects are dependent on the β-catenin pathway and occur despite an apparent downregulation of the mTOR pathway (Fig 9).

Pharmacological inhibition of GSK3-β with SB216763 significantly induced LEC migration, proliferation and, more impressively, tubulation *in vitro*. The specificity of this compound for GSK3-β and its lack of effects on GSK3-α [38] is unclear and therefore we confirmed our results with a novel inhibitor recently shown to selectively block GSK3-β and not GSK3-α kinase activity [37]. Furthermore, siRNA-induced silencing of GSK3-β showed similar effects on LEC network formation *in vitro*. To ascertain that interference with GSK3-β activity would lead to lymphangiogenesis through β-catenin dependent pathways, LECs were subjected to siRNA-mediated silencing of β-catenin. This resulted in loss of response to the stimulatory effects of pharmacological inhibition of GSK3-β. These results highlight the importance of β-catenin in lymphangiogenesis *in vitro*.

Our findings contrast with previous reports on the effects of lithium chloride a commonly used and non-specific GSK3-β inhibitor on lymphangiogenesis *in vivo* [39]. However, inhibition of lymphangiogenesis was attributed to the effects of lithium chloride on cancer cell production of TGF-β. The direct effects of GSK3-β-inhibition on LECs independently of TGF-β were not studied.

To investigate mechanisms of lymphangiogenesis driven by GSK3-β inhibition, we also interrogated the mTOR pathway. GSK3-β has previously been shown to phosphorylate PTEN at residue Ser362 and Thr366 [40]. Phosphorylation at residue 366 is destabilizing, resulting in decreased PTEN levels [31]. Here we found that GSK3-β inhibition led to PTEN dephosphorylation at residue T366 and its subsequent stabilization, with downstream inactivation of AKT and pS6Kinase. The effects of GSK3-β on the mTOR pathway are controversial, with one study showing that GSK3-β negatively regulates the mTOR pathway [17], and another demonstrating that GSK3 positively regulated S6Kinase [41]. These effects could therefore be cell type-specific. Regardless, these effects on the mTOR pathway were surprising since mTOR is a known downstream target of many lymphangiogenic growth factors and rapamycin is a potent inhibitor of lymphangiogenesis [22–25].

GSK3-β is ubiquitously expressed, with extensive targets [42]; thus, systemic delivery would therefore result in unwanted adverse effects and concerns over malignancy driven by its activation of the β-catenin pathway. Local delivery either through hydrogels, nanoparticles or antibodies directly targeting cell surface receptors of LEC could be of potential therapeutic benefits, and in the case of lung disease, consideration could be given to airway delivery leading to local deposition in the lung parenchyma [43].

To conclude, in this study, GSK3-β has been shown to regulate lymphangiogenesis. Inhibition of GSK3-β with a small molecule or with siRNA induced lymphangiogenesis through β-catenin-dependent pathways. Pre-clinical studies targeting this pathway are necessary to establish the merit of this strategy *in vivo*.

## Supporting information

S1 Fig Full uncut gels. (DOCX)

## References

[1] Adamczyk LA, Gordon K, Kholova I, Meijer-Jorna LB, Telinius N, Gallagher PJ, et al Lymph vessels: the forgotten second circulation in health and disease. Virchows Arch. 2016;469(1):3–17. 10.1007/s00428-016-1945-6 . 27173782 PMC4923112

[2] Farnsworth RH, Achen MG, Stacker SA. The evolving role of lymphatics in cancer metastasis. Curr Opin Immunol. 2018;53:64–73. 10.1016/j.coi.2018.04.008 . 29698919

[3] Astin JW, Jamieson SM, Eng TC, Flores MV, Misa JP, Chien A, et al An in vivo antilymphatic screen in zebrafish identifies novel inhibitors of mammalian lymphangiogenesis and lymphatic-mediated metastasis. Mol Cancer Ther. 2014;13(10):2450–62. 10.1158/1535-7163.MCT-14-0469-T . 25053822

[4] Schulz MM, Reisen F, Zgraggen S, Fischer S, Yuen D, Kang GJ, et al Phenotype-based high-content chemical library screening identifies statins as inhibitors of in vivo lymphangiogenesis. Proc Natl Acad Sci U S A. 2012;109(40):E2665–74. 10.1073/pnas.1206036109 . 22949700 PMC3479568

[5] Kalin RE, Banziger-Tobler NE, Detmar M, Brandli AW. An in vivo chemical library screen in Xenopus tadpoles reveals novel pathways involved in angiogenesis and lymphangiogenesis. Blood. 2009;114(5):1110–22. 10.1182/blood-2009-03-211771 . 19478043 PMC2721788

[6] Klotz L, Norman S, Vieira JM, Masters M, Rohling M, Dube KN, et al Cardiac lymphatics are heterogeneous in origin and respond to injury. Nature. 2015;522(7554):62–7. 10.1038/nature14483 . 25992544 PMC4458138

[7] Cui Y, Liu K, Monzon-Medina ME, Padera RF, Wang H, George G, et al Therapeutic lymphangiogenesis ameliorates established acute lung allograft rejection. J Clin Invest. 2015;125(11):4255–68. 10.1172/JCI79693 . 26485284 PMC4639995

[8] Baluk P, Tammela T, Ator E, Lyubynska N, Achen MG, Hicklin DJ, et al Pathogenesis of persistent lymphatic vessel hyperplasia in chronic airway inflammation. J Clin Invest. 2005;115(2):247–57. 10.1172/JCI22037 . 15668734 PMC544601

[9] Stump B, Cui Y, Kidambi P, Lamattina AM, El-Chemaly S. Lymphatic Changes in Respiratory Diseases: More than Just Remodeling of the Lung? Am J Respir Cell Mol Biol. 2017;57(3):272–9. 10.1165/rcmb.2016-0290TR . 28443685 PMC5625224

[10] Wong BW, Zecchin A, Garcia-Caballero M, Carmeliet P. Emerging Concepts in Organ-Specific Lymphatic Vessels and Metabolic Regulation of Lymphatic Development. Dev Cell. 2018;45(3):289–301. . 29738709 10.1016/j.devcel.2018.03.021

[11] Coso S, Bovay E, Petrova TV. Pressing the right buttons: signaling in lymphangiogenesis. Blood. 2014;123(17):2614–24. 10.1182/blood-2013-12-297317 . 24608974

[12] Zheng W, Aspelund A, Alitalo K. Lymphangiogenic factors, mechanisms, and applications. J Clin Invest. 2014;124(3):878–87. 10.1172/JCI71603 . 24590272 PMC3934166

[13] Choi I, Lee S, Kyoung Chung H, Suk Lee Y, Eui Kim K, Choi D, et al 9-cis retinoic acid promotes lymphangiogenesis and enhances lymphatic vessel regeneration: therapeutic implications of 9-cis retinoic acid for secondary lymphedema. Circulation. 2012;125(7):872–82. 10.1161/CIRCULATIONAHA.111.030296 . 22275501 PMC3327127

[14] Greco KV, Lara PF, Oliveira-Filho RM, Greco RV, Sudo-Hayashi LS. Lymphatic regeneration across an incisional wound: inhibition by dexamethasone and aspirin, and acceleration by a micronized purified flavonoid fraction. Eur J Pharmacol. 2006;551(1–3):131–42. 10.1016/j.ejphar.2006.08.090 . 17045986

[15] Beurel E, Grieco SF, Jope RS. Glycogen synthase kinase-3 (GSK3): regulation, actions, and diseases. Pharmacol Ther. 2015;148:114–31. 10.1016/j.pharmthera.2014.11.016 . 25435019 PMC4340754

[16] Wu D, Pan W. GSK3: a multifaceted kinase in Wnt signaling. Trends Biochem Sci. 2010;35(3):161–8. . 19884009 10.1016/j.tibs.2009.10.002 PMC2834833

[17] Inoki K, Ouyang H, Zhu T, Lindvall C, Wang Y, Zhang X, et al TSC2 integrates Wnt and energy signals via a coordinated phosphorylation by AMPK and GSK3 to regulate cell growth. Cell. 2006;126(5):955–68. . 16959574 10.1016/j.cell.2006.06.055

[18] Cha B, Geng X, Mahamud MR, Fu J, Mukherjee A, Kim Y, et al Mechanotransduction activates canonical Wnt/beta-catenin signaling to promote lymphatic vascular patterning and the development of lymphatic and lymphovenous valves. Genes Dev. 2016;30(12):1454–69. 10.1101/gad.282400.116 . 27313318 PMC4926867

[19] Muley A, Odaka Y, Lewkowich IP, Vemaraju S, Yamaguchi TP, Shawber C, et al Myeloid Wnt ligands are required for normal development of dermal lymphatic vasculature. PLoS One. 2017;12(8):e0181549 10.1371/journal.pone.0181549 . 28846685 PMC5573294

[20] Cha B, Geng X, Mahamud MR, Zhang JY, Chen L, Kim W, et al Complementary Wnt Sources Regulate Lymphatic Vascular Development via PROX1-Dependent Wnt/beta-Catenin Signaling. Cell Rep. 2018;25(3):571–84 e5. . 30332639 10.1016/j.celrep.2018.09.049 PMC6264919

[21] Petrova TV, Makinen T, Makela TP, Saarela J, Virtanen I, Ferrell RE, et al Lymphatic endothelial reprogramming of vascular endothelial cells by the Prox-1 homeobox transcription factor. EMBO J. 2002;21(17):4593–9. 10.1093/emboj/cdf470 . 12198161 PMC125413

[22] Huber S, Bruns CJ, Schmid G, Hermann PC, Conrad C, Niess H, et al Inhibition of the mammalian target of rapamycin impedes lymphangiogenesis. Kidney Int. 2007;71(8):771–7. . 17299523 10.1038/sj.ki.5002112

[23] Patel V, Marsh CA, Dorsam RT, Mikelis CM, Masedunskas A, Amornphimoltham P, et al Decreased lymphangiogenesis and lymph node metastasis by mTOR inhibition in head and neck cancer. Cancer Res. 2011;71(22):7103–12. 10.1158/0008-5472.CAN-10-3192 . 21975930 PMC3443559

[24] Baluk P, Yao LC, Flores JC, Choi D, Hong YK, McDonald DM. Rapamycin reversal of VEGF-C-driven lymphatic anomalies in the respiratory tract. JCI Insight. 2017;2(16). 10.1172/jci.insight.90103 . 28814666 PMC5621869

[25] Luo Y, Liu L, Rogers D, Su W, Odaka Y, Zhou H, et al Rapamycin inhibits lymphatic endothelial cell tube formation by downregulating vascular endothelial growth factor receptor 3 protein expression. Neoplasia. 2012;14(3):228–37. . 22496622 10.1593/neo.111570 PMC3323900

[26] Cui Y, Liu K, Lamattina AM, Visner G, El-Chemaly S. Lymphatic Vessels: The Next Frontier in Lung Transplant. Ann Am Thorac Soc. 2017;14(Supplement_3):S226–S32. 10.1513/AnnalsATS.201606-465MG . 28945468 PMC5711339

[27] El-Chemaly S, Malide D, Zudaire E, Ikeda Y, Weinberg BA, Pacheco-Rodriguez G, et al Abnormal lymphangiogenesis in idiopathic pulmonary fibrosis with insights into cellular and molecular mechanisms. Proc Natl Acad Sci U S A. 2009;106(10):3958–63. 10.1073/pnas.0813368106 . 19237567 PMC2646625

[28] Bruyere F, Noel A. Lymphangiogenesis: in vitro and in vivo models. FASEB J. 2010;24(1):8–21. 10.1096/fj.09-132852 . 19726757

[29] Carpentier G. Contribution: Angiogenesis Analyzer. ImageJ News, 5 10 2012 2012.

[30] Saxton RA, Sabatini DM. mTOR Signaling in Growth, Metabolism, and Disease. Cell. 2017;168(6):960–76. . 28283069 10.1016/j.cell.2017.02.004 PMC5394987

[31] Maccario H, Perera NM, Davidson L, Downes CP, Leslie NR. PTEN is destabilized by phosphorylation on Thr366. Biochem J. 2007;405(3):439–44. 10.1042/BJ20061837 . 17444818 PMC2267318

[32] Memmott RM, Dennis PA. Akt-dependent and -independent mechanisms of mTOR regulation in cancer. Cell Signal. 2009;21(5):656–64. 10.1016/j.cellsig.2009.01.004 . 19166931 PMC2650010

[33] Ikeda S, Kishida S, Yamamoto H, Murai H, Koyama S, Kikuchi A. Axin, a negative regulator of the Wnt signaling pathway, forms a complex with GSK-3beta and beta-catenin and promotes GSK-3beta-dependent phosphorylation of beta-catenin. EMBO J. 1998;17(5):1371–84. 10.1093/emboj/17.5.1371 . 9482734 PMC1170485

[34] Wagner FF, Bishop JA, Gale JP, Shi X, Walk M, Ketterman J, et al Inhibitors of Glycogen Synthase Kinase 3 with Exquisite Kinome-Wide Selectivity and Their Functional Effects. ACS Chem Biol. 2016;11(7):1952–63. 10.1021/acschembio.6b00306 . 27128528

[35] Coghlan MP, Culbert AA, Cross DA, Corcoran SL, Yates JW, Pearce NJ, et al Selective small molecule inhibitors of glycogen synthase kinase-3 modulate glycogen metabolism and gene transcription. Chem Biol. 2000;7(10):793–803. . 11033082 10.1016/s1074-5521(00)00025-9

[36] Chelko SP, Asimaki A, Andersen P, Bedja D, Amat-Alarcon N, DeMazumder D, et al Central role for GSK3beta in the pathogenesis of arrhythmogenic cardiomyopathy. JCI Insight. 2016;1(5). 10.1172/jci.insight.85923 . 27170944 PMC4861310

[37] Wagner FF, Benajiba L, Campbell AJ, Weiwer M, Sacher JR, Gale JP, et al Exploiting an Asp-Glu "switch" in glycogen synthase kinase 3 to design paralog-selective inhibitors for use in acute myeloid leukemia. Sci Transl Med. 2018;10(431). 10.1126/scitranslmed.aam8460 . 29515000 PMC6553635

[38] Lal H, Ahmad F, Woodgett J, Force T. The GSK-3 family as therapeutic target for myocardial diseases. Circ Res. 2015;116(1):138–49. 10.1161/CIRCRESAHA.116.303613 . 25552693 PMC4283216

[39] Maeng Y-S, Lee R, Lee B, Choi S-i, Kim EK. Lithium inhibits tumor lymphangiogenesis and metastasis through the inhibition of TGFBIp expression in cancer cells. Scientific Reports. 2016;6:20739 https://www.nature.com/articles/srep20739#supplementary-information. 26857144 10.1038/srep20739 PMC4746585

[40] Al-Khouri AM, Ma Y, Togo SH, Williams S, Mustelin T. Cooperative phosphorylation of the tumor suppressor phosphatase and tensin homologue (PTEN) by casein kinases and glycogen synthase kinase 3beta. J Biol Chem. 2005;280(42):35195–202. 10.1074/jbc.M503045200 . 16107342

[41] Shin S, Wolgamott L, Yu Y, Blenis J, Yoon SO. Glycogen synthase kinase (GSK)-3 promotes p70 ribosomal protein S6 kinase (p70S6K) activity and cell proliferation. Proc Natl Acad Sci U S A. 2011;108(47):E1204–13. 10.1073/pnas.1110195108 . 22065737 PMC3223461

[42] Sutherland C. What Are the bona fide GSK3 Substrates? Int J Alzheimers Dis. 2011;2011:505607 10.4061/2011/505607 . 21629754 PMC3100594

[43] El-Sherbiny IM, El-Baz NM, Yacoub MH. Inhaled nano- and microparticles for drug delivery. Glob Cardiol Sci Pract. 2015;2015:2 10.5339/gcsp.2015.2 . 26779496 PMC4386009

