# Supplementary material for: Glycogen synthase kinase 3-β inhibition induces lymphangiogenesis through β-catenin-dependent and mTOR-independent pathways
Source: PLoS One. 2019 Apr 9;14(4):e0213831. doi: 10.1371/journal.pone.0213831 (PMC6456176; doi:10.1371/journal.pone.0213831)
Supplement: S1 Fig — (DOCX) [file pone.0213831.s001.docx]

Original western blot images uncut.

**Figure 3**

**Figure 3A**


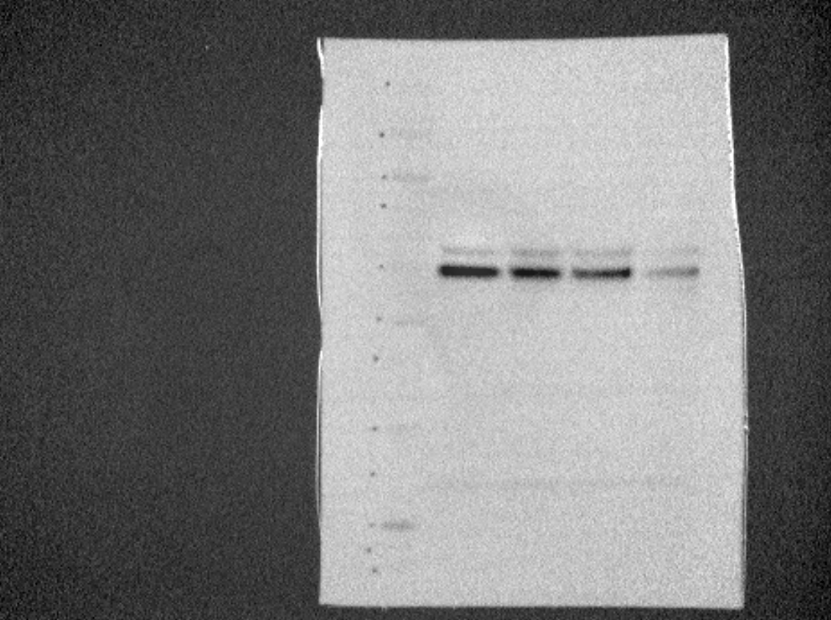


p-P70S6K

SB216763 - +


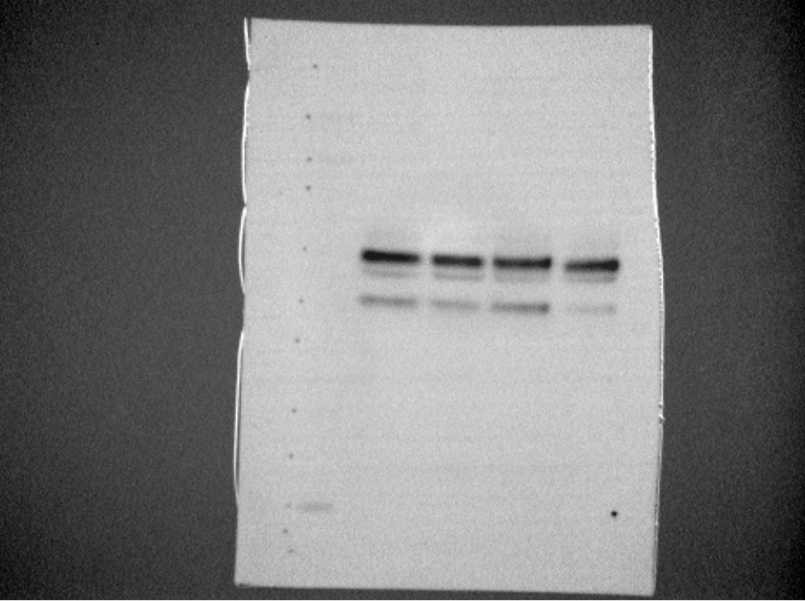


P70S6K

SB216763 - +


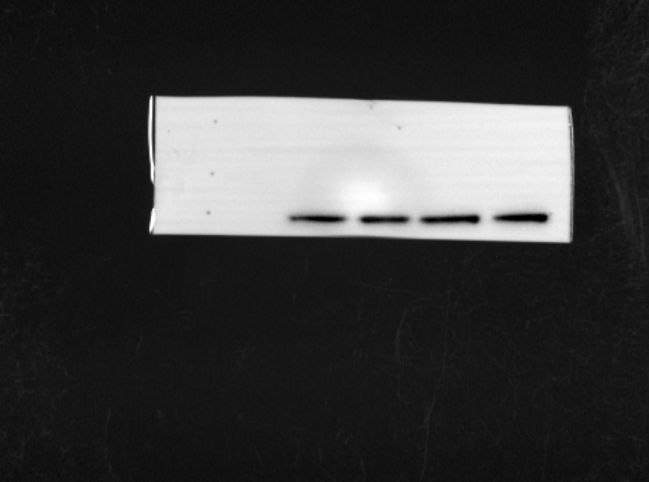


β- actin

**Figure 3B**


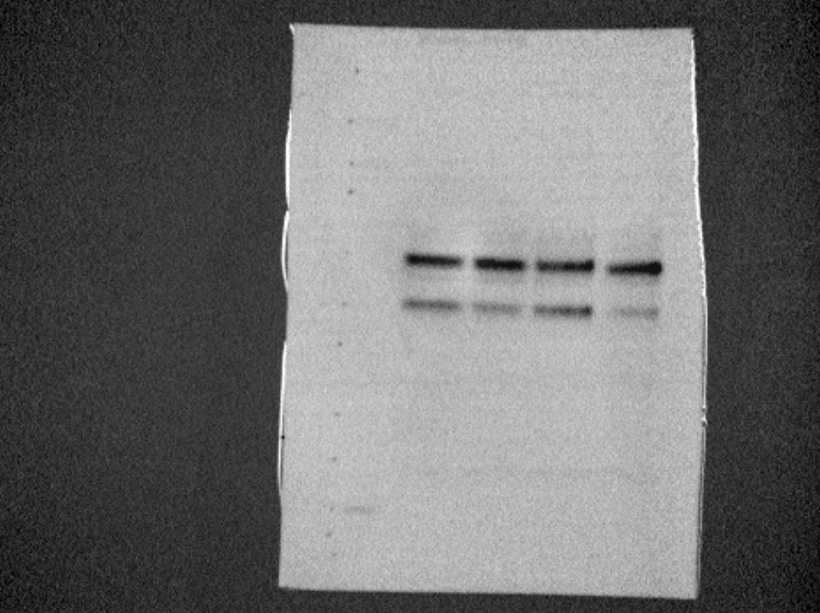


SB216763 - +

p-PTEN

SB216763 - +


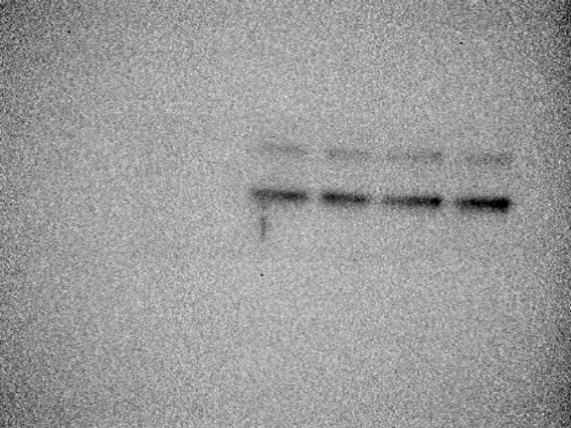


PTEN

**Figure 3C**

p-P70S6

SB216763 - +


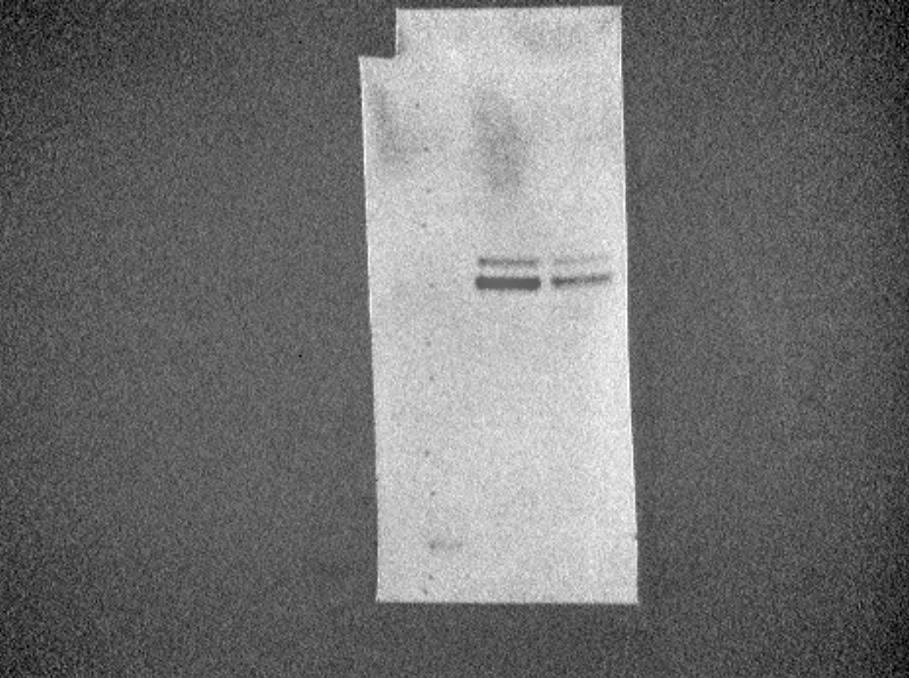


p-P70S6K


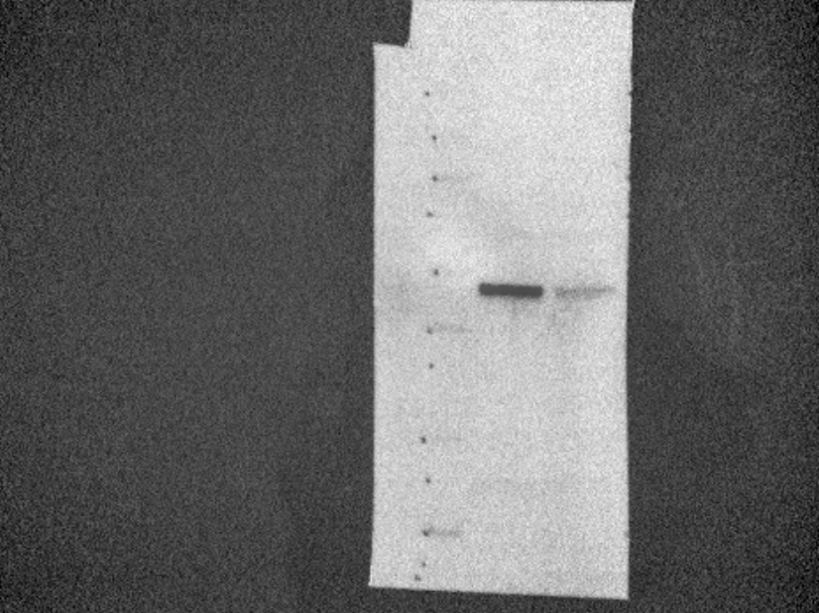


p-Akt

SB216763 - +

Total AKT


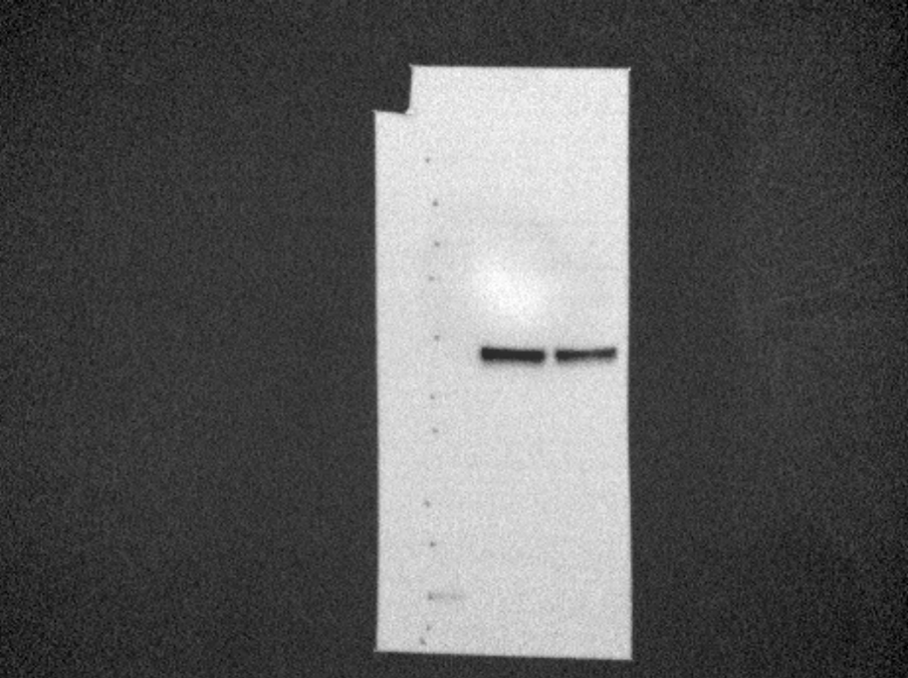


Total Akt

SB216763 - +


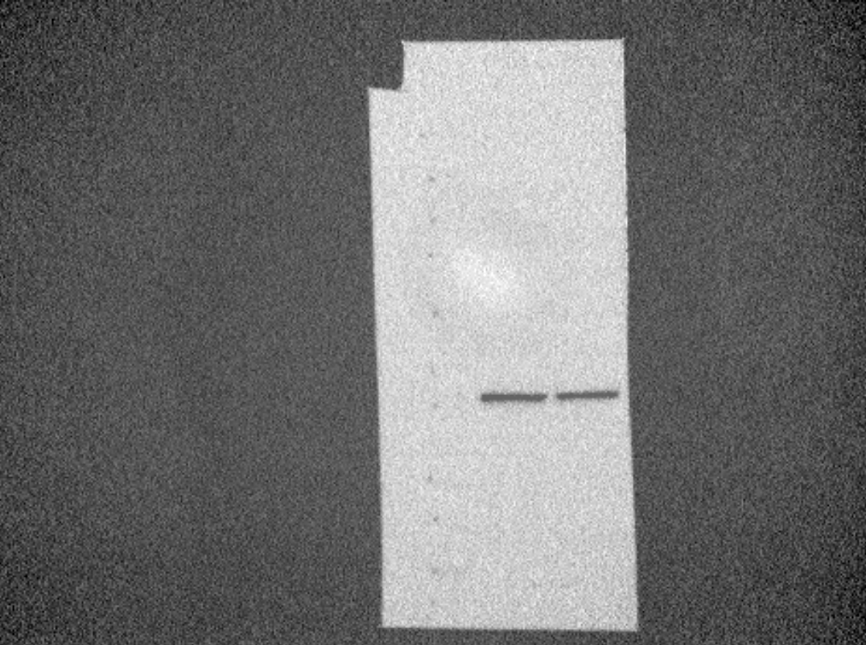


SB216763 - +

β-actin

**Figure 4**

SB216763 - +

Lane cut out from Figure 4A- Experimental condition was full media,

which in context of serum starvation in lanes 1 and 3 was difficult to interpret

and not included in manuscript.


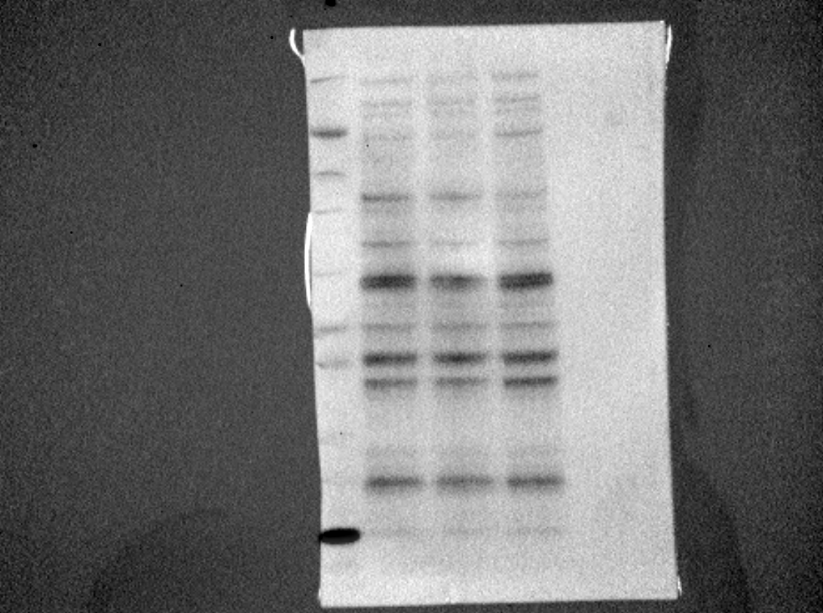


SB216763 - +

p-β-catenin


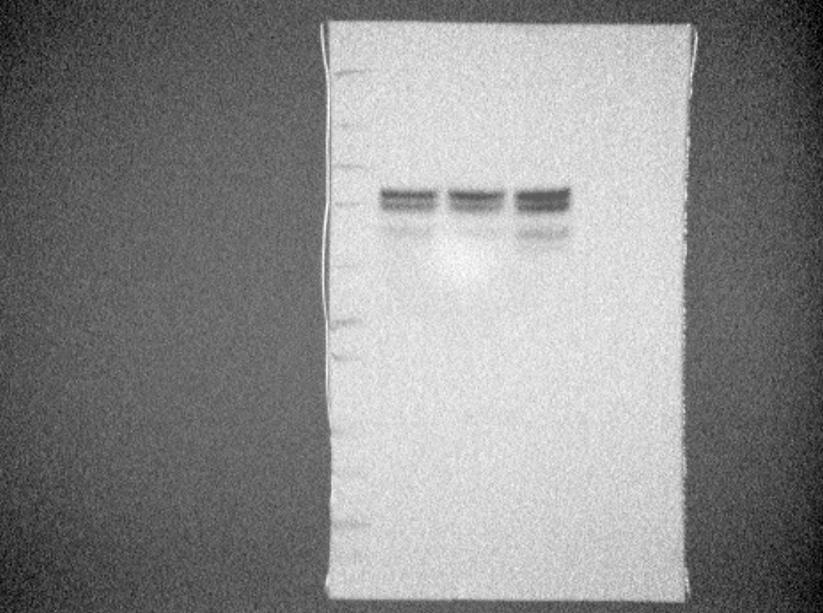


β-catenin


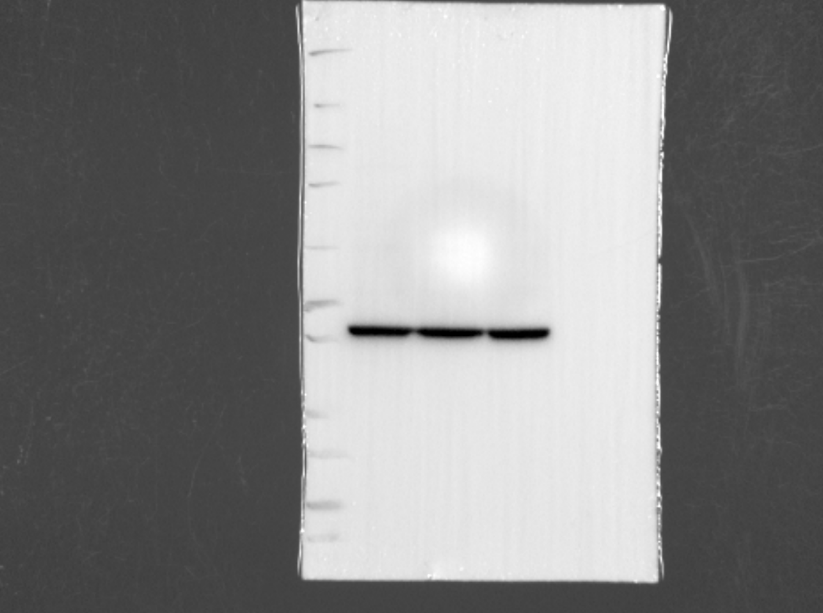


β-Actinn

SB216763 - +

**Figure 5**

**Figure 5A**


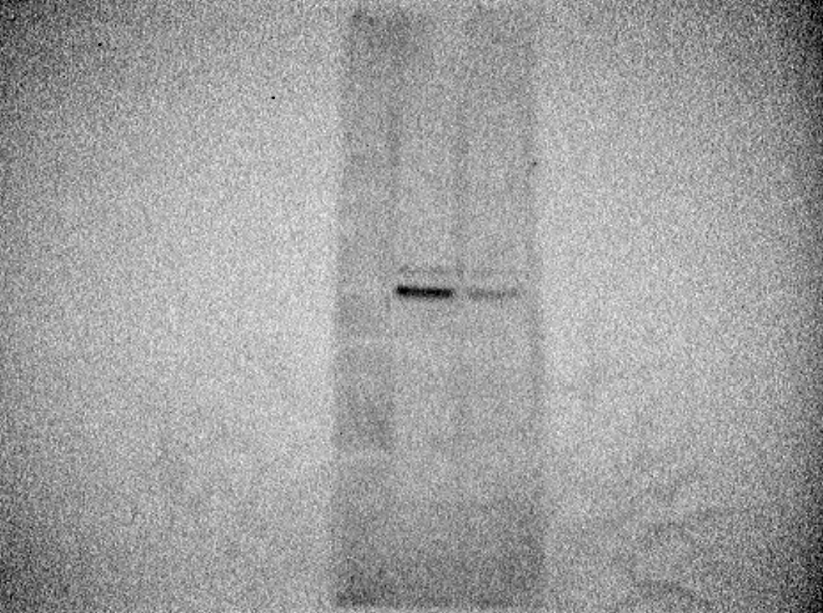


BRD 3731 - +

p-P70S6K


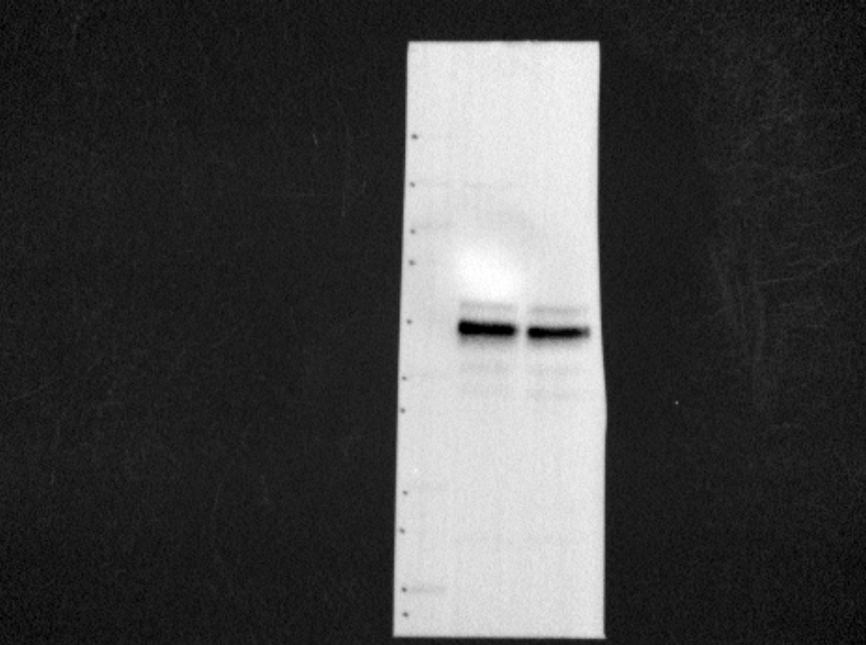


BRD 3731 - +

Total P70S6K


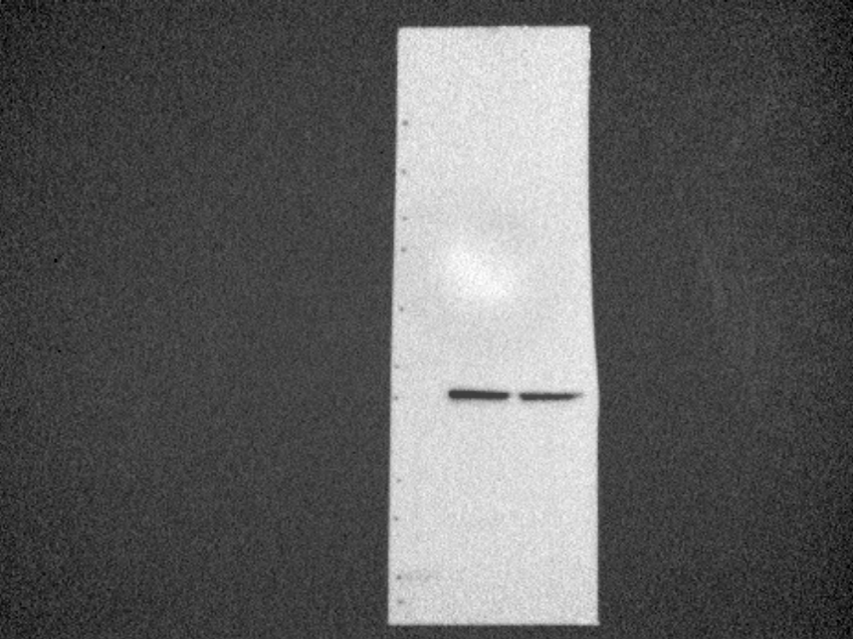


BRD 3731 - +

β-Actin

**Figure 5B**


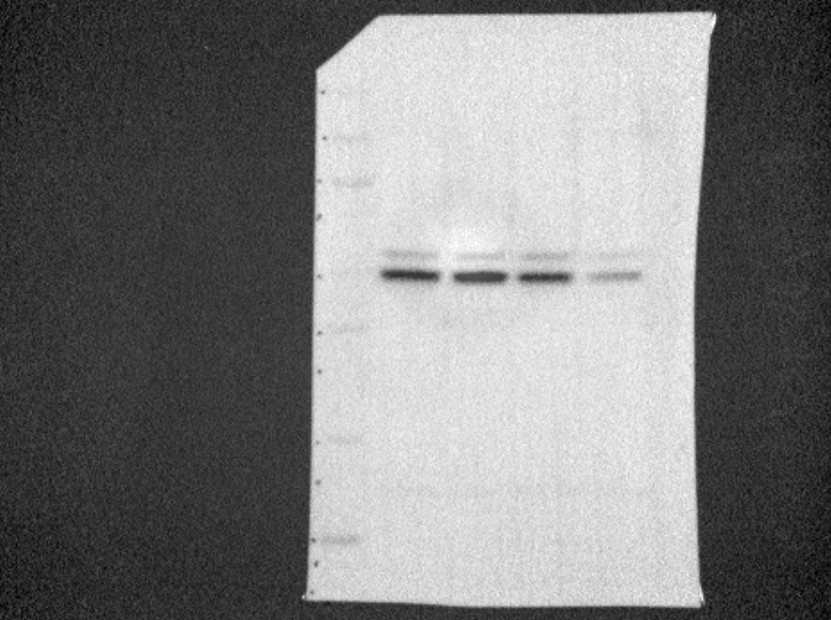


p-P70S6K

BRD 3731 - +

p-PTEN


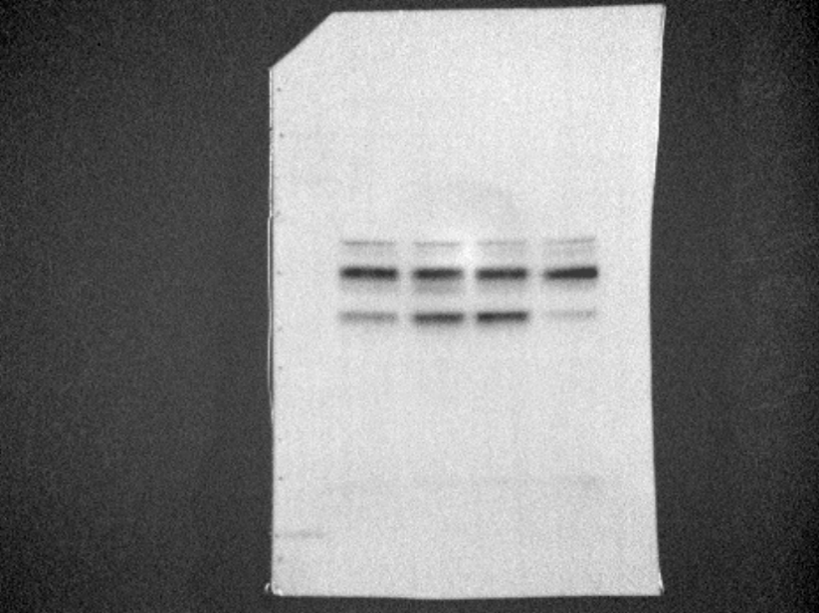


BRD 3731 - +

p-PTEN


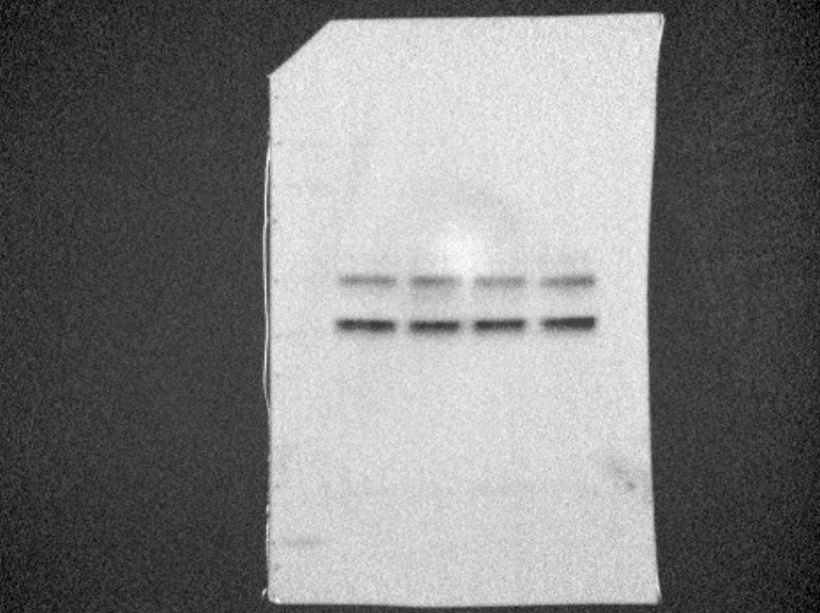


BRD 3731 - +

PTEN


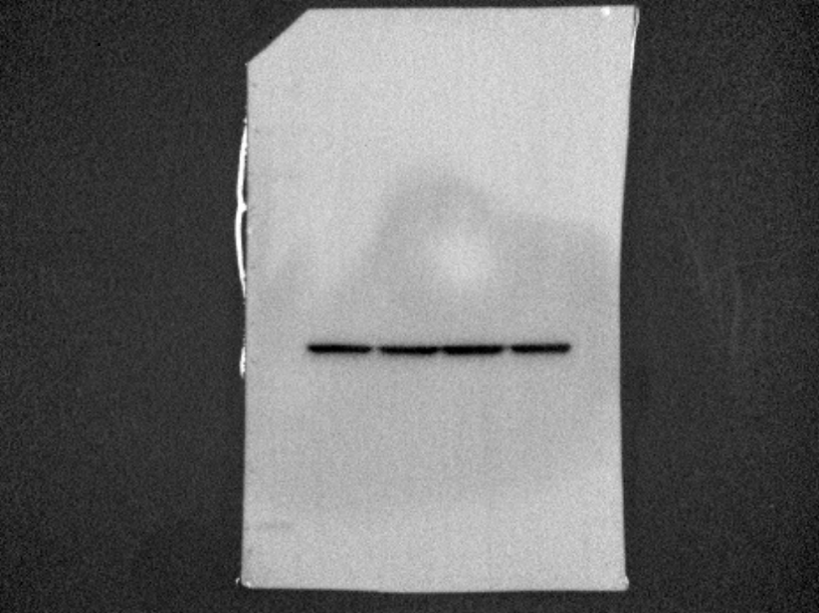


β-actin

BRD 3731 - +

**Figure 5C**


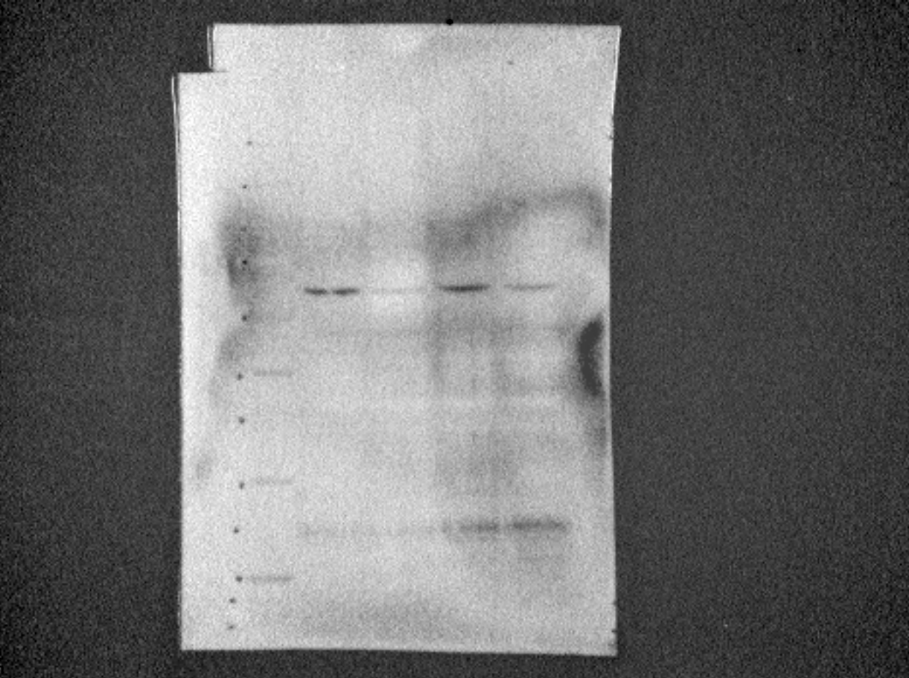


p-β-catenin

BRD 3731 - +


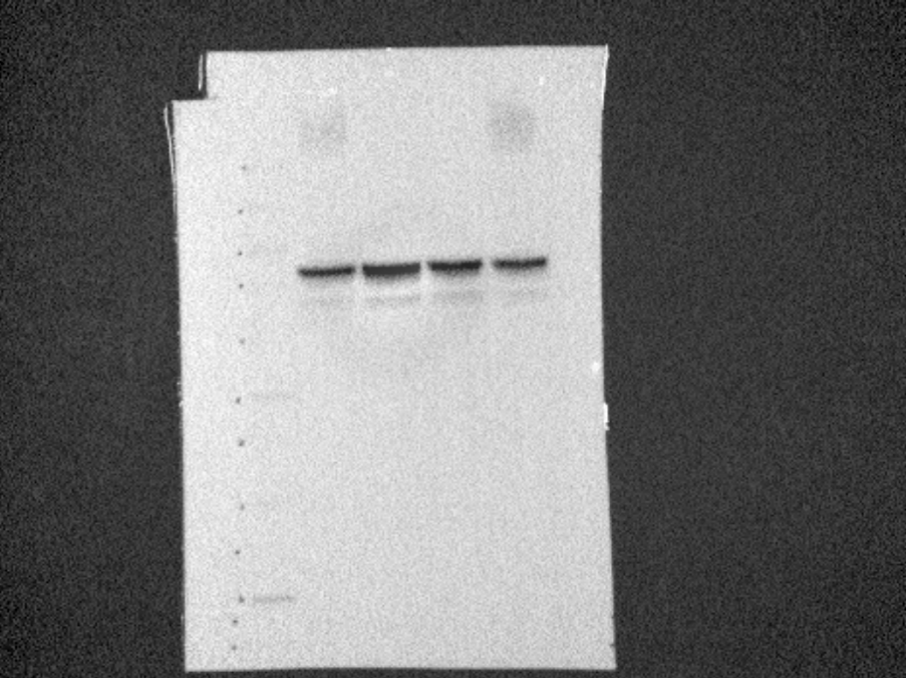


BRD 3731 - +

β-catenin


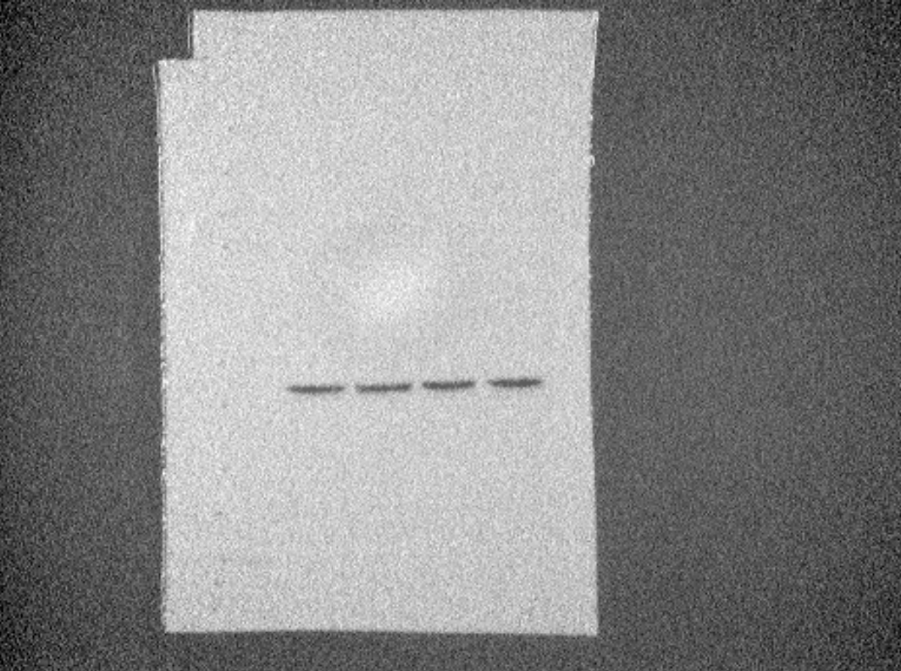


BRD 3731 - +

β-actin

**Figure 6**

**
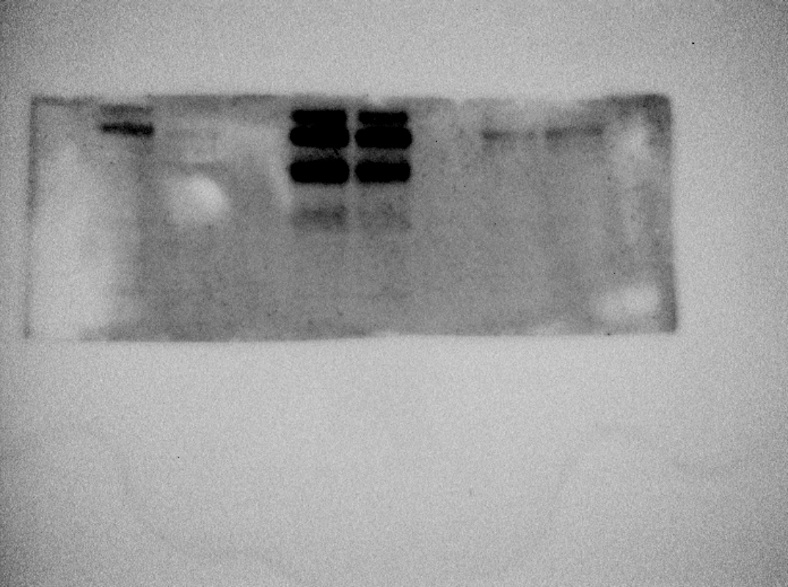
**

**
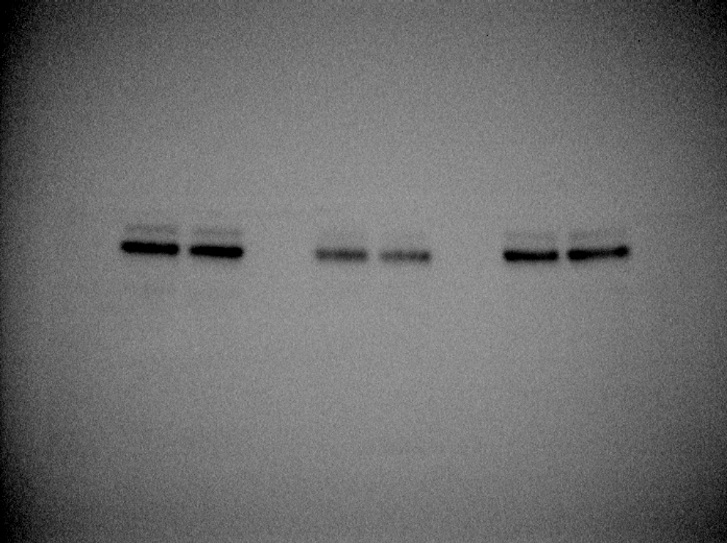
**

**
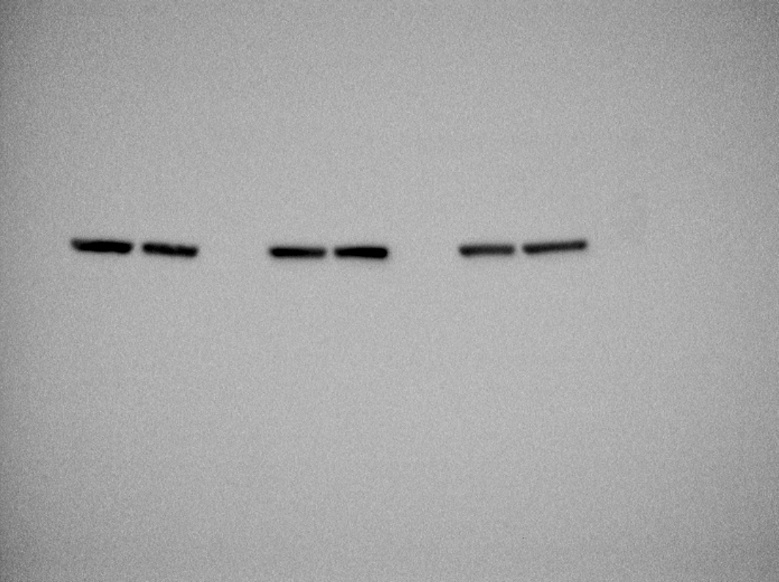
**

**p-PTEN**

**
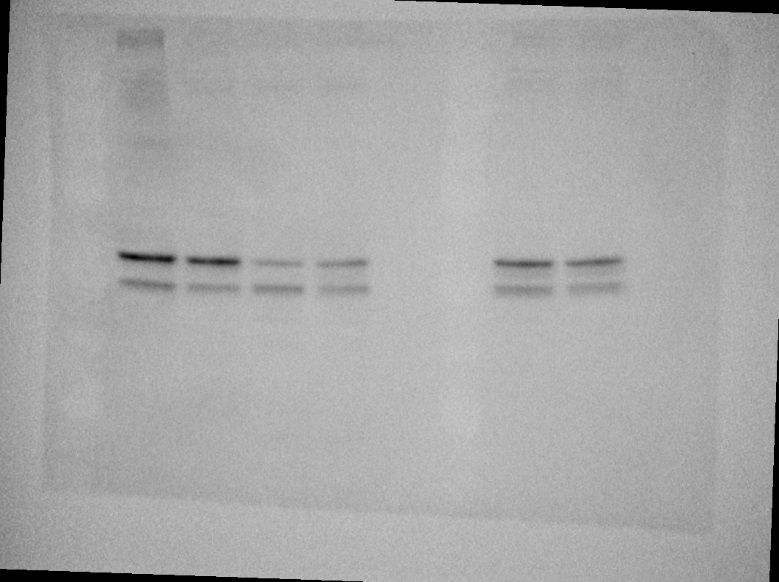
**

**PTEN**

**
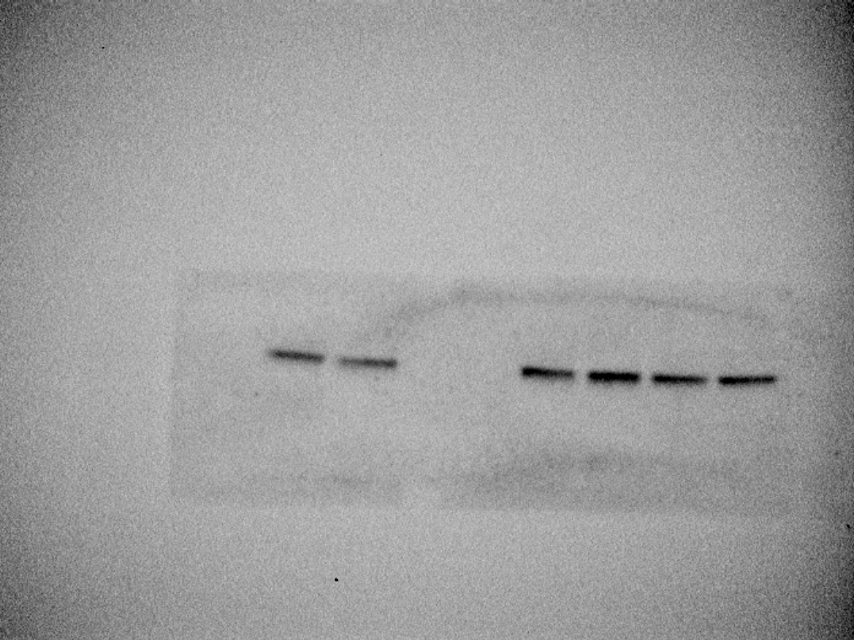
**

**GSK3-B**

**
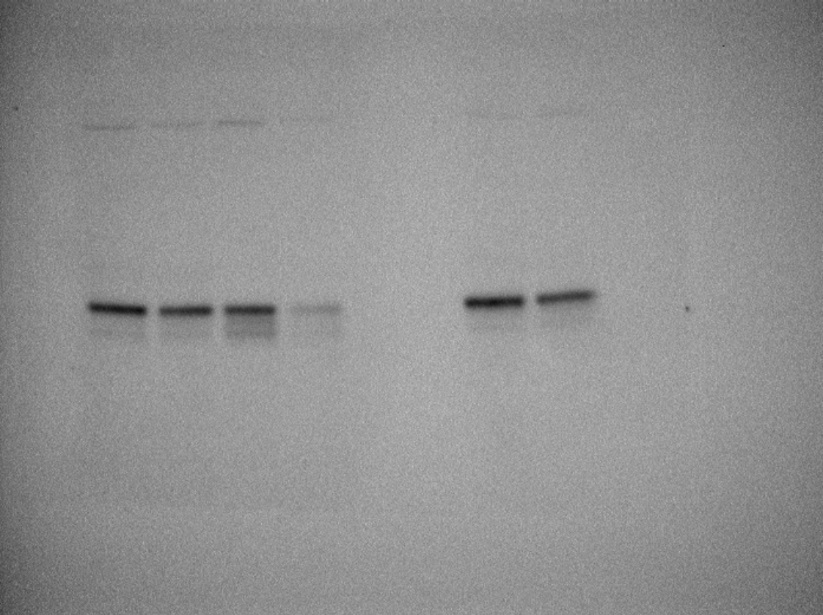
**

**B-actin**

**
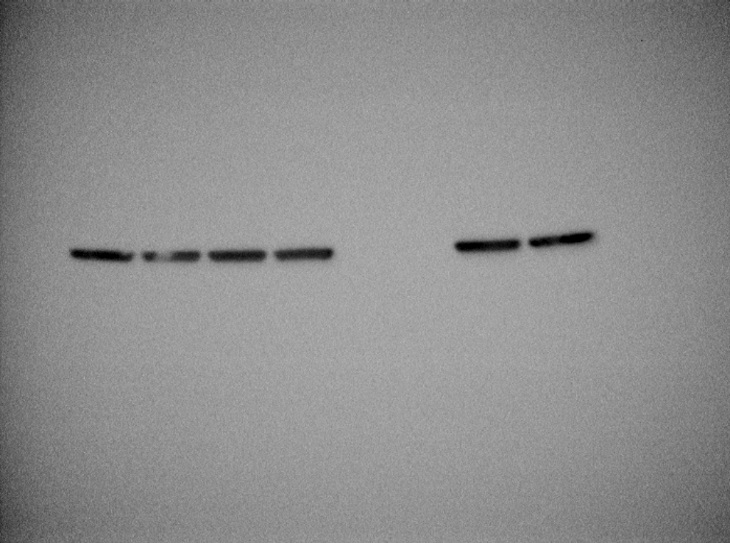
**

**B-catenin**

**
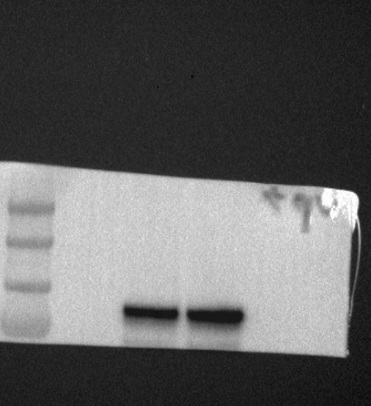
**

**p-B-catenin**

**
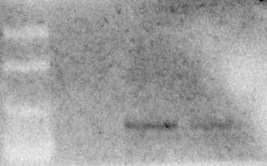
**

**b-actin**

**
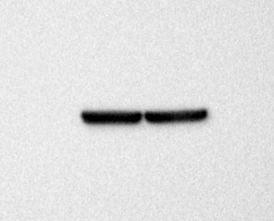
**

**GSK3-B**

**
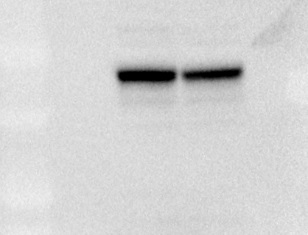
**

**Figure 7**

Lipofectamine - + - -

Control siRNA - - + -

GSK3-β siRNA - - - +

**
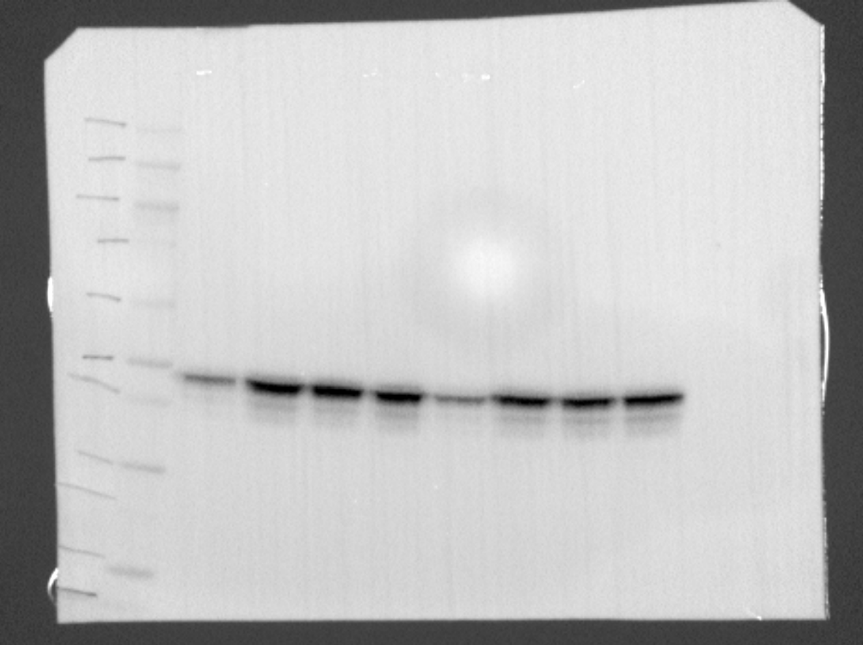
**

GSK3-β

**
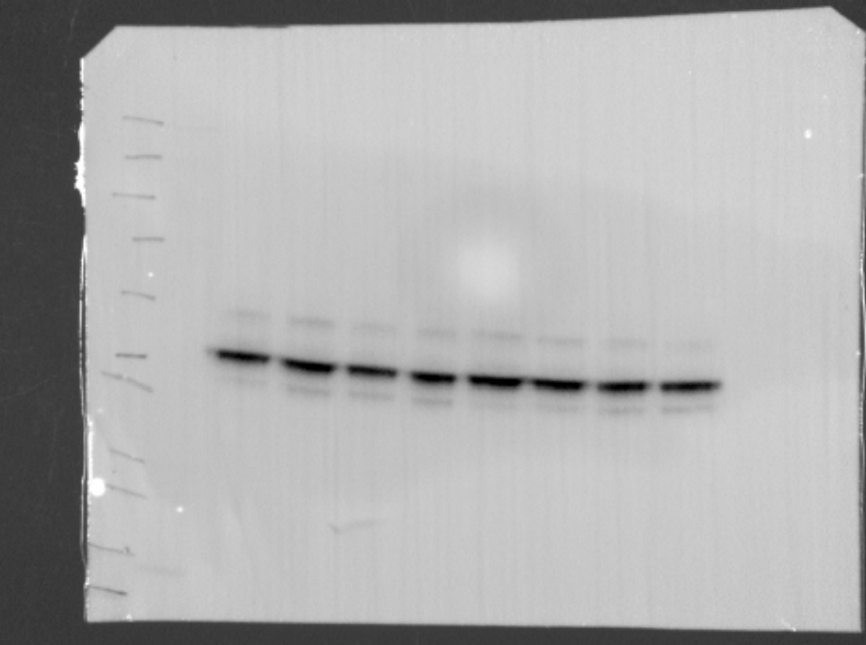
**

GSK3-α

**
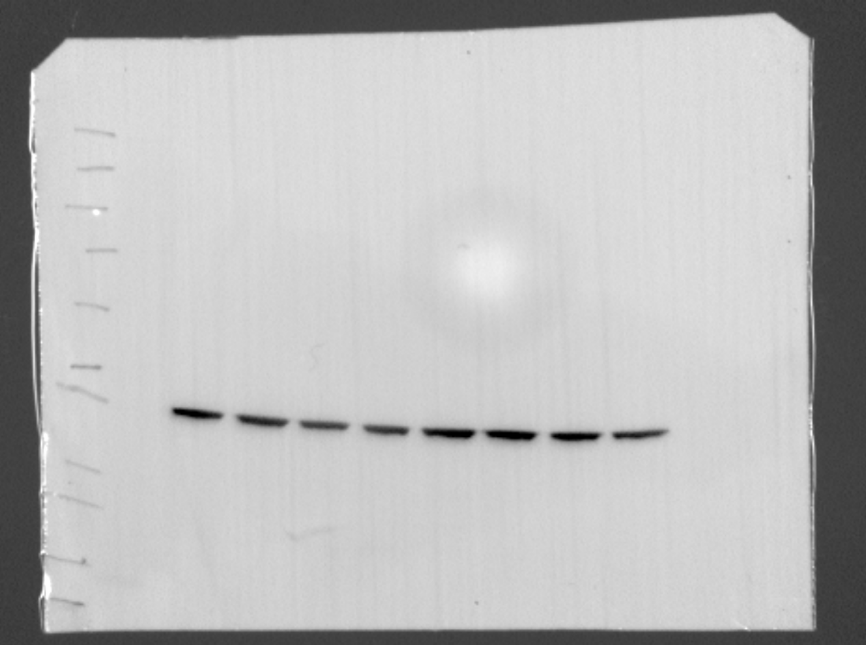
**

β-actin

**Figure 8**

Lipofectamine - + - -

Control siRNA - - + -

Β-catenin siRNA - - - +

**
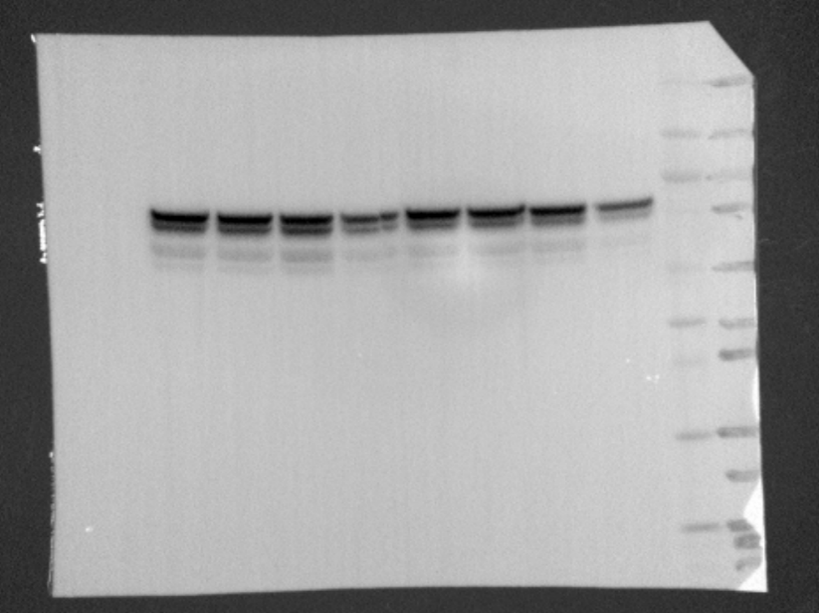
**

β-catenin

**
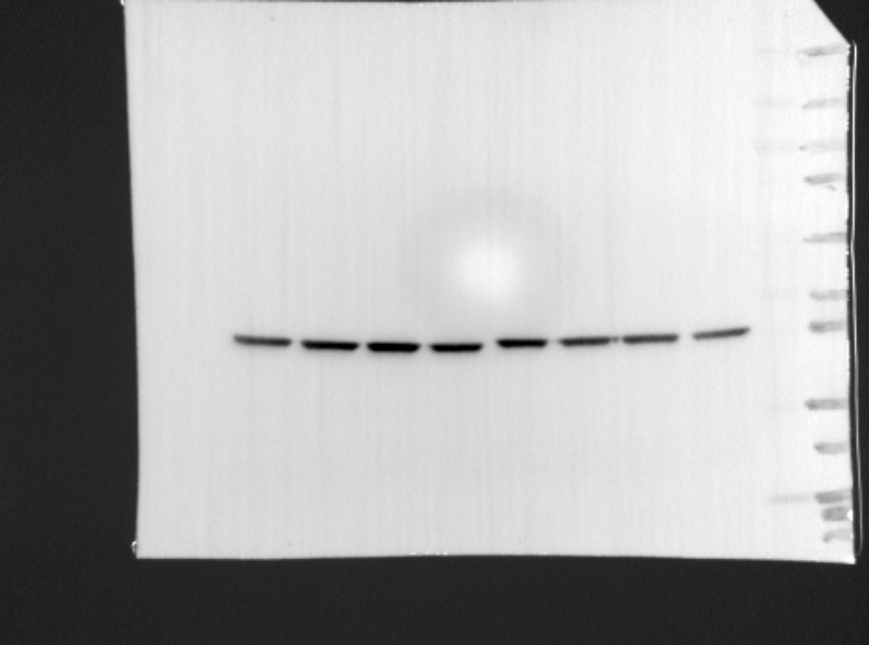
**

β-actin
